# New Aromatic Bisabolane Derivatives with Lipid-Reducing Activity from the Marine Sponge *Myrmekioderma* sp.

**DOI:** 10.3390/md17060375

**Published:** 2019-06-22

**Authors:** Margarida Costa, Laura Coello, Ralph Urbatzka, Marta Pérez, Margret Thorsteinsdottir

**Affiliations:** 1 Faculty of Pharmaceutical Sciences, University of Iceland, Hofsvallagata 53, 107 Reykjavik, Iceland; margreth@hi.is; 2 Research & Development Department, PharmaMar S.A., Pol. Ind. La Mina Norte, Avda. de los Reyes 1, 28770 Colmenar Viejo (Madrid), Spain; lcoello@pharmamar.com (L.C.); mperez@pharmamar.com (M.P.); 3 Interdisciplinary Centre of Marine and Environmental Research (CIIMAR/CIMAR), University of Porto, Avenida General Norton de Matos, s/n, 4450-208 Matosinhos, Portugal; ralph.urbatzka.ciimar@gmail.com

**Keywords:** marine sponges, natural compounds, bisabolane-related compounds, bioactivity, obesity, whole small animal models

## Abstract

The previously reported 1-(2,4-dihydroxy-5-methylphenyl)ethan-1-one (**1**), (1’Z)-2-(1’,5’-dimethylhexa-1’,4’-dieny1)-5-methylbenzene-1,4-diol (**2**), and 1,8-epoxy-1(6),2,4,7,10-bisaborapentaen-4-ol (**5**) together with four new structures of aromatic bisabolane-related compounds (**3**, **4**, **6**, **7**) were isolated from the marine sponge *Myrmekioderma* sp. Compounds **1**, **2**, and **5** were identified based on spectral data available in the literature. The structures of the four new compounds were experimentally established by 1D and 2D-NMR and (−)-HRESIMS spectral analysis. Cytotoxic and lipid-reducing activities of the isolated compounds were evaluated. None of the isolated compounds were active against the tested cancer cell lines; however, lipid-reducing activity was found for compounds **2**–**5** and **7** in the zebrafish Nile red fat metabolism assay. This class of compounds should be further explored for their suitability as possible agents for the treatment of lipid metabolic disorders and obesity.

## 1. Introduction

Marine organisms are exposed to continuous and strong selection pressures due to the huge variations in predation, temperature, pressure, and light. For these reasons, they are known to produce secondary metabolites as a mechanism of defense [1]. These secondary metabolites represent an impressive source of structurally diverse molecules with biological activities which can lead to major advances in the field of medicinal chemistry [2,3].

Among marine organisms, sponges represent a prolific source of a vast number of diverse molecules with potential applications for human health. The numbers of compounds isolated from sponges have been increasing every year [4]. Among these compounds, marine sesquiterpenes are recognized as an important class with great structural diversity and a wide range of bioactivities such as anti-HIV, antitumor, antibiotic, antiviral, cytotoxic, insecticidal, antifeedant, and antifungal activities [5,6]. Bisabolane compounds constitute a class of sesquiterpene bioactive metabolites that have been identified from both terrestrial plants and marine invertebrates [7,8]. Several bioactivities are associated with this class of compounds, such as cytotoxicity [9,10] and antifungal [10] properties. Furthermore, their suitability for use as biodiesel is also under investigation [11].

Obesity is increasing at epidemic rates and new therapeutics are needed in order to prevent and control this disorder [12]. Scientists have been working hard to find new compounds from different natural sources, both terrestrial and marine, that show anti-obesity activity [13,14,15]. Several marine secondary metabolites with anti-obesity properties have already been reported, such as the 5-alkylpyrrole-2-carboxaldehyde derivatives, isolated from the sponge *Mycale lissochela*, which have protein-tyrosine phosphatase 1B (a recognized target for obesity) inhibitory activity [6]. Also, citreorosein and questinol, isolated from the marine sponge-associated fungus *Talaromyces stipitatus* KUFA 0207, decreased the neutral lipids in the zebrafish Nile red fat metabolism assay [16].

As a part of our on-going screening program for the discovery of new secondary metabolites from marine sponges, the study of an organic extract of *Myrmekioderma* sp. resulted in the isolation of seven natural compounds: three known compounds 1-(2,4-dihydroxy-5-methylphenyl)ethan-1-one (**1**), (1’Z)-2-(1’,5’-dimethylhexa-1’,4’-dieny1)-5-methylbenzene-1,4-diol (**2**), 1,8-epoxy-1(6),2,4,7,10-bisaborapentaen-4-ol (**5**), and four new bisabolane derivatives (**3**, **4**, **6** and **7**). Their planar structures were fully elucidated using spectroscopic and spectrometric techniques. All compounds were tested for their cytotoxic and lipid-reducing activities. Compounds **2**, **5**, and **7** were highly active in the zebrafish Nile red fat metabolism assay and compounds **3** and **4** showed moderate activity in the same bioassay. Cytotoxic activity in the four cancer cell lines tested was not observed for any of the isolated compounds.

## 2. Results and Discussion

### Isolation and Structure Elucidation

**Figure 1:**
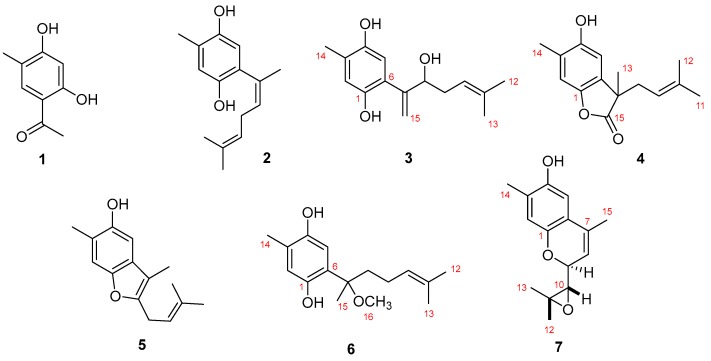
Chemical structures of the compounds **1**–**7** isolated from *Myrmekioderma* sp.

The sponge *Myrmekioderma* sp. was collected by hand while scuba diving in Boano (Indonesia). The specimen was repeatedly extracted using dichloromethane:methanol (1:1 v/v). The crude organic extract was subsequently partitioned between *n*-hexane, ethyl acetate, *n*-butanol, and water. The *n*-hexane and ethyl acetate fractions, after vacuum liquid chromatography (VLC) and semi-preparative reverse-phase HPLC separations, led to the isolation of the seven pure compounds shown in Figure 1.

Compound **1** was isolated as a dark-brown oil. It was identified as 1-(2,4-dihydroxy-5-methylphenyl)ethan-1-one, as shown in Figure 1, based on spectral data available in the literature [17]. 

Also based on spectral data available in the literature, compound **2** was identified as (1’Z)-2-(1’,5’-dimethylhexa-1’,4’-dieny1)-5-methylbenzene-1,4-diol [18]. 

**Figure 2:**
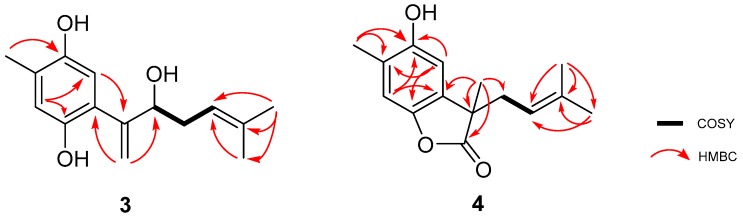
Key ^1^H-^1^H COSY and HMBC correlations of compounds **3** and **4**.

Compound **3** was isolated as a yellow amorphous solid. The molecular formula C_15_H_20_O_3_ was established based on the (−)-HRESIMS molecular ion *m/z* 247.1344 [M − H]^−^ (calculated 247.1334), which imposed six degrees of unsaturation. The ^13^C-NMR spectrum of **3,** compiled in Table 1, confirmed the presence of fifteen carbon signals which were assigned, by DEPT and HMQC spectral analysis, to two tertiary (δ_C_ 26.1, 18.1) and one secondary (δ_C_ 15.8) methyls, two methylenes (δ_C_ 120.3, 34.3) of which one was double bonded (δ_C_ 120.3), two aromatic (δ_C_ 118.8, 117.2), one double-bonded (δ_C_ 118.8), and one hydroxylated (δ_C_ 76.7) methine and six non-protonated carbons (δ_C_ 148.0, 147.5, 147.0, 136.9, 125.4, 124.2). From the listed non-protonated carbons, two were hydroxylated (δ_C_ 148.0, 147.5). In accordance, the ^1^H-NMR spectrum exhibited three methyl singlets (δ_H_ 2.20, 1.71, 1.53), two splitting methylenes (δ_H_ 5.43 and 5.24, 2.30, and 2.15), the first two suggesting a double bond, and four methines (δ_H_ 6.68, 6.50, 5.06, 4.40). Based on COSY and HMBC spectral data, as shown in Figure 2, a simple sesquiterpene structure was proposed for this compound. ^1^H and ^13^C data, together with the H-2 HMBC correlations with C-1 and C-5 revealed the presence of a tetrasubstituted benzene ring. C-1 and C-4 deshielded carbon resonances (δ_C_ 147.5, 147.0) indicated the presence of a benzene-1,4-diol. Me-14 was assigned based on the HMBC correlation Me-14/C-4 and C-6 substitution based on the correlations H-5/C-7 and H_2_-15/C-6. The double bond, suggested by H_2_-15 resonances (δ_C_ 120.3, δ_H_ 5.43, 5.24), was elucidated based on the previously described HMBC correlation of H_2_-15/C-6 with the hydroxylated C-8 (δ_C_ 76.7). The COSY correlations H-8/H_2_-9 and H_2_-9/H-10 allowed assignment of the methylene and Δ^10(11)^ double bond. The HMBC cross signals of the methyl groups Me-12 and Me-13 with each other and of both of them with C-10 and C-11 completed the structure. Unfortunately, a paucity of material prevented the assignment of the C-8 absolute stereochemistry. Thus, the structure of **3** was elucidated as the curcuhydroquinone derivative shown in Figure 1: 6-(3-hydroxy-6-methyl-1,5-heptadien-2-yl)-3-methylbenzene-1,4-diol.

Compound **4** was isolated as a yellow amorphous powder. The molecular formula C_15_H_18_O_3_ was calculated based on the (−)-HRESIMS *m/z* 245.1126 [M − H]^−^ (calculated 245.1177) molecular ion peak indicating the existence of seven degrees of unsaturation. Compound **4** ^1^H and ^13^C-NMR spectral data, compiled in Table 1, resembled those of compounds **2** and **3**. The ^13^C-NMR spectrum confirmed the presence of fifteen carbon signals which were assigned, by DEPT and HMQC spectral analysis, to four methyls (δ_C_ 25.8, 23.5, 18.0, 16.2), one methylene (δ_C_ 37.4), two aromatic (δ_C_ 112.5, 110.0), and one olefinic (δ_C_ 117.2) methines and seven non-protonated carbons (δ_C_ 180.8, 150.4, 146.4, 136.5, 130.4, 123.9, 48.1), of which two were hydroxylated (δ_C_ 150.4, 146.4) and one an ester (δ_C_ 180.8). In accordance, the ^1^H-NMR spectrum showed four singlet methyls (δ_H_ 2.26, 1.60, 1.56, 1.44), one splitting methylene (δ_H_ 2.58, 2.42), two aromatic (δ_H_ 6.87, 6.64), and one olefinic (δ_H_ 4.85) methine. The same tetrasubstituted benzene ring found in compounds **2** and **3** was also proposed for compound **4** due to the similarity of the ^1^H and ^13^C-NMR data. The HMBC correlations H-2/C-4, H-2/C-6, H-5/C-1, H-5/C-3, and H-5/C-4 confirmed the proposed sub-structure. Further HMBC correlations Me-14/C-3 and Me-14/C-4 corroborated the assignment of this methyl group. The most notable new features of compound **4** were the carbonyl resonance (δ_C_ 180.8) and a non-protonated alkane carbon (δ_C_ 48.1). Allocation of these was accomplished based on the HMBC correlations of Me-13 with C-6, C-7, C-8, and C-15, confirming a lactone sub-structure. The COSY correlation H_2_-8/H-9 allowed assignment of the methylene and the Δ^9(10)^ double bond, which was linked to the non-protonated C-10 based on the HMBC correlations of Me-11 and Me-12 with both C-9 and C-10. As a result, the structure of compound **4** was elucidated as the sesquiterpene shown in Figure 1: 4-hydroxy-3,7-dimethyl-7-(3-methylbut-2-en-1-yl)benzofuran-15-one. 

Compound **5** was isolated as a yellow amorphous powder. Spectral data available in the literature allowed its identification as 1,8-epoxy-1(6),2,4,7,10-bisaborapentaen-4-ol [19].

**Figure 3:**
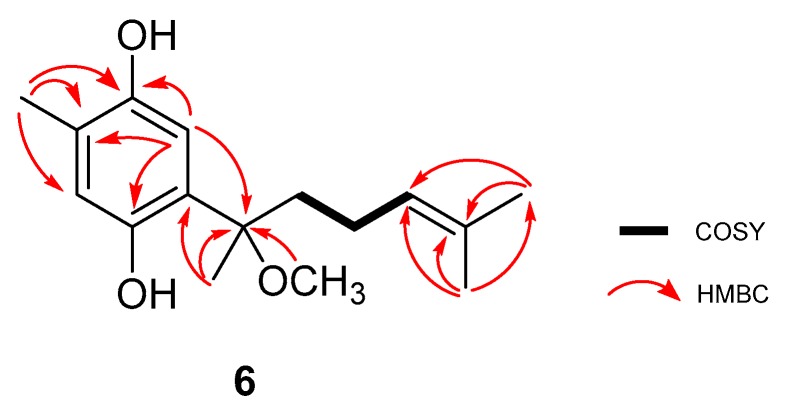
Key ^1^H-^1^H COSY, HMBC, and TOCSY correlations of compound **6**.

Compound **6** was isolated as dark-brown oil. The molecular formula C_16_H_24_O_3_ was established based on the (−)-HRESIMS *m*/*z* molecular ion peak 263.1610 [M − H]^−^ (calculated 263.1647), indicating five degrees of unsaturation. Both ^1^H and ^13^C-NMR indicated structural similarities with compounds **2**–**5** (Table 2). The same tetrasubstituted hydroquinone ring found in compounds **2** and **3** was suggested for compound **6.** The HMBC correlations H-5/C-1, H-5/C-3, and H-5/C-4, represented in Figure 3, confirmed the proposed hydroquinone ring. The methyl-14 substitution was assigned based on HMBC correlations of this group with C-2, C-3, and C-4. The C-6 substitution was confirmed based on the HMBC correlations H-5/C-7, Me-15/C-6, and Me-15/C-7. The last correlation, together with Me-16/C-7, provided the key to methyl groups -15 and -16. The ^13^C-NMR and DEPT data suggested the presence of two methylenes (δ_C_ 39.8, 22.8), consistent with a side chain one carbon longer than found previously. Me-12 and Me-13 were assigned based on their HMBC correlations between each other and with C-10 and C-11. The COSY correlation H-10/H-9 allowed completion of this second sub-structure.

The configuration of the chiral center present in compound **6** could not be clearly elucidated with the material available and the physio-chemical information obtained for this compound. Thus, the structure of **6** was elucidated as the curcuhydroquinone derivative 6-(2-methoxy-6-methylhept-5-en-2-yl)-3-methylbenzene-1,4-diol (Figure 1).

**Figure 4:**
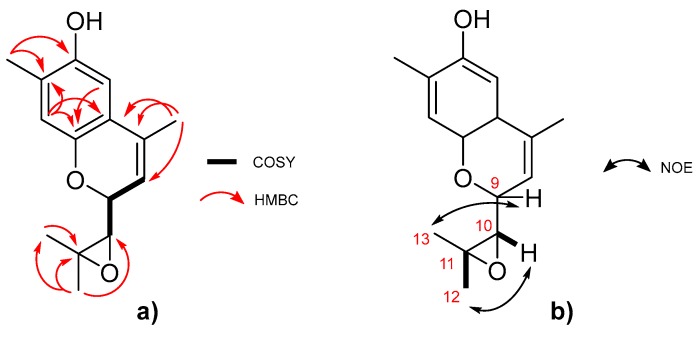
Key correlations for the elucidation of compound **7**. (**a**) ^1^H-^1^H COSY and HMBC. (**b**) NOESY (partial structure).

Compound **7** was isolated as a green crystal. The (−)-HRESIMS showed the molecular ion peak *m/z* 245.1126 [M − H]^−^ (calculated 245.1177), very similar to the one reported for compound **4**. As for compound **4**, C_15_H_18_O_3_ was the calculated molecular formula, indicating the existence of seven degrees of unsaturation. Analysis of the ^1^H and ^13^C-NMR spectral data, compiled in Table 2, and a comparison with of the data for the previously elucidated compounds revealed the presence of the phenolic part of the structure, but with considerable modifications in the side chain. As seen in Figure 4a, the HMBC correlations Me-15/C-6 and Me-15/C-7, together with the deshielded resonance of C-7 (δ_C_ 122.0) allowed assignment of this methyl group and the Δ^7(8)^ double bond. Based on the COSY correlations between H-8, H-9, and H-10, a spin system was defined. Chemical shifts of the positions 9 (δ 4.51/55.6) and 10 (δ 3.06/63.8) indicated they were bearing an oxygen atom. The HMBC correlations between Me-15 with C-7/C-8/C-9 and H-10 C-11/Me-12/Me-13 allowed us to establish the position of the side chain. Furthermore, the chemical shift of the quaternary C-11 (δC 57.7) indicated that it was also oxygenated. This fact, along with the molecular formula, suggests cyclization of the phenol OH to the C-9 position and an epoxide between C-10 and C-11. Additionally, the chemical shift of the epoxide positions supported the proposed structure when compared to other related epoxide fragments described in the literature [20,21]. Finally, the MS fragmentation pattern showing the *m/z* fragments 230.1421, 165.0497, and 122.0332 (see Supplementary Materials Figure S45) reinforced this proposal.

A comparison of the resonances, together with a large coupling constant between H-9 and H-10 (8.3 Hz), allowed the relative configuration at C-9/C-10 to be determined as anti [22]. Furthermore, the NOE correlations H-12/H-10 and H-13/H-9, represented in Figure 4b, confirmed this configuration. Thus, the structure of **7** was elucidated as the curcuphenol derivative shown in Figure 1: 9-(3,3-dimethyloxiran-2-yl)-1,7-dimethyl-7-chromen-4-ol.

Several bisabolane-type sesquiterpenoids have been reported from different marine organisms, such as the marine sponge *Halichondria* sp. [18], the gorgonians *Pseudopterogorgia* spp. [7] or the red algae *Laurencia scoparia* [23]. The isolation of bisabolane-related compounds from microorganisms, such as the marine-derived fungus *Aspergillus* sp. [24], has been used to suggest that these compounds are produced by microbial-associated organisms and not directly by the host. In this work, we were able to isolate four new bisabolane-related compounds. For these new compounds from this work, no assumptions can be made as to whether the producer is the sponge or possible associated microorganisms since the metabolites were extracted indistinctly.

Bisabolane-like compounds have previously been isolated from marine sponges [9,10]. However, to the best of our knowledge, this work represents the first report of this class of compounds from *Myrmekioderma* sp. Besides belonging to a known class of compounds, the four new isolated bisabolane-related metabolites show novel structural features. Cyclic bisabolane and metabolites bearing oxo functionality are both uncommon among this group of compounds, highlighting the importance of these discoveries. 

From the biosynthetic point of view, bisabolane-related compounds have already been described as a result of the combination of the shikimic and mevalonic acid pathways [25,26] and the same routes are proposed for the described compounds. Compound **4**, however, has a migrated carbon relative to the curcuphenol skeleton, a feature that can be found in other related-compounds [27]. Compound **4** is, therefore, proposed to be obtained from tetraketide 3-methyl-orsellinic acid [27,28]. As such, there is strong evidence that the compounds originate from a fungi-associated strain. All seven isolated compounds were tested for their cytotoxic activity against A-549 human lung carcinoma cells, MDA-MB-231 human breast adenocarcinoma cells, HT-29 human colorectal carcinoma, and PSN1 human pancreatic adenocarcinoma cells. Compounds **1**–**7** were inactive (IC_50_ > 20 μM) in all the cancer cell lines tested.

**Figure 5:**
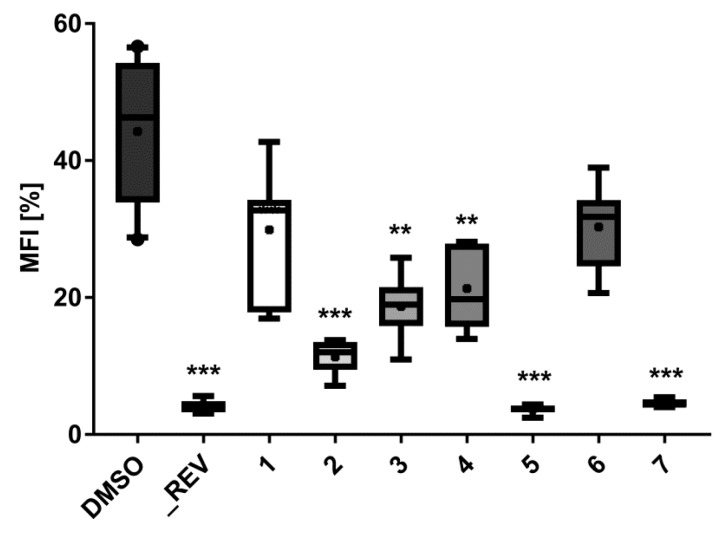
Lipid-reducing activity of compounds **1**–**7** in zebrafish Nile red fat metabolism assay. MFI represents the mean fluorescence intensity, indicative of neutral lipids. A solvent control with 0.1% DMSO was included in the bioassay, together with the positive control 50 µM resveratrol (REV). Per treatment, 6–8 individual zebrafish larvae were used. ** *p* < 0.01, *** *p* < 0.001.

**Figure 6:**
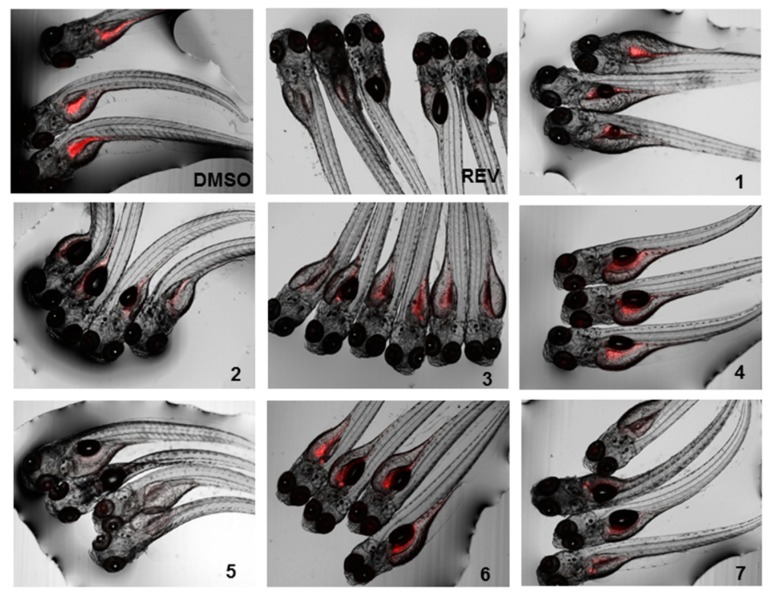
Representative images of the zebrafish Nile red assay. Images show the overlay of the fluorescence and bright field images; 0.1% DMSO was used as solvent control and 50 µM resveratrol (REV) as positive control.

The lipid-reducing activity of compounds **1**–**7** was also tested using the zebrafish Nile red fat metabolism assay (Figure 5 and Figure 6). This whole small animal model was already successfully used in the discovery of lipid-reducing compounds from fungus [16], chemically modified polyphenols [29] and cyanobacteria [30], and offers higher physiological relevance compared to commonly used cellular in vitro models. Furthermore, it was previously shown that zebrafish larvae responded similarly to humans if challenged with known lipid regulator drugs [31]. The results showed that compounds **2**, **5**, and **7** have potent lipid-reducing activity (IC_50_ = 1.78, 0.84, and 1.22 µM, respectively), reducing significantly the zebrafish Nile red fluorescence intensity, which is indicative of the total amount of neutral lipids. Compounds **3** and **4** also showed moderate lipid-reducing activity (IC_50_ = 7.89, 12.61 µM, respectively). None of the compounds **1**–**7** had any general toxicity on zebrafish larvae and additionally did not cause any malformations. It is interesting to observe that all the active compounds are bisabolane-related, while compound **1** did not show activity. The structural differences between compounds **2** or **3** compared to compound **6** caused the inactivation of the compound, but cyclizing the side chain (compound **7**) did not. Therefore, more studies are needed in order to understand the relationship between the chemical structure and its lipid-reducing activity.

## 3. Materials and Methods 

### 3.1. General Experiments

Optical rotations were measured on a Jasco P-1020 polarimeter. The UV spectra were measured using an Agilent 8453 UV-vis spectrometer. The IR spectra were recorded on a Nicolet iZ10 (ThermoFisher Scientific) FTIR spectrophotometer. The NMR experiments were performed on a Bruker 400 spectrometer at 400/100 MHz (^1^H/^13^C). Chemical shifts were reported in ppm using residual CD_3_OD (*δ* 3.31 for ^1^H and 49.0 for ^13^C) and CDCl_3_ (*δ* 7.26 for ^1^H and 77.2 for ^13^C) as internal references. The HRESIMS was performed on a Waters Synapt G1 UPLC-QTOF mass spectrometer in negative ionization mode.

### 3.2. Biological Sample

The *Myrmekioderma* sp. sponge was collected by hand while scuba diving in Boano (Indonesia). The sponge was immediately frozen and kept under these conditions until extraction. The specimen was identified by María Jesús Uriz at CEAB, Blanes, Spain. A voucher specimen (ORMA135834) is deposited at PharmaMar facilities (Madrid, Spain).

### 3.3. Extraction and Isolation

The frozen sponge specimen (320 g) was repeatedly extracted using dichloromethane: methanol (CH_2_:Cl_2_:MeOH 1:1 v/v). The extract was concentrated under vacuum to yield 25.91 g of crude extract. This crude extract was dissolved in 300 mL of water and subsequently extracted with *n*-hexane (3 × 300 mL), ethyl acetate (EtOAc) (3 × 300 mL), and butanol (*n*-BuOH) (2 × 250 mL). The *n*-hexane extract (6.11 g) was subjected to reversed phase VLC over RP-18 silica gel with a stepped gradient from H_2_O:MeOH (3:1 v/v) to dichloromethane (CH_2_Cl_2_). Fraction 1 (95.6 mg), eluted with H_2_O:MeOH (3:1 v/v), was subjected to semi-preparative HPLC (Gemini-NX C18 110A, Phenomenex, 5µ, 10.0 × 250 mm, gradient H_2_O:MeCN 60:40 v/v to 50:50 v/v, in 15 min, 3 mL/min) to yield compound **1** (6.4 mg) at 10 min. Fraction 3 (1640.7 mg), eluted with pure MeOH, was initially separated by preparative HPLC (Luna C18 100A, Phenomenex, 5 µ, 21.20 × 250 mm, gradient H_2_O:MeCN (25:75 v/v) to 0:100, in 30 min, 6 mL/min), yielding HPLC Fraction 2 at 14 minutes (444.2 mg). This fraction was again separated by preparative HPLC (Luna C18 100A, Phenomenex, 5 µ, 21.20 × 250 mm, gradient H_2_O:MeCN 50:50 v/v to 40:60 v/v, in 25 min, 10 mL/min), yielding compound **2** (98.6 mg) at 21 minutes and HPLC Fraction 4 at 24 minutes (146.6 mg). The HPLC Fraction 4 was submitted to a final semi-preparative HPLC separation (Gemini-NX C18 110A column, 5 µ, Phenomenex, 10.0 × 250 mm, gradient H_2_O:MeCN 50:50 v/v to 30:70 v/v, in 35 min, 2.3 mL/min) to yield compounds **3** (1.3 mg) at 11 min, **4** (4.9 mg) at 21 min and **5** (9.4 mg) at 34 min. The EtOAc extract from the original liquid/liquid extraction was also subjected to reversed phase VLC over RP-18 silica gel with a stepped gradient from H_2_O:MeOH (3:1 v/v) to CH_2_Cl_2_. Fraction 2 (1021.7 mg) eluted with H_2_O:MeOH (1:3 v/v) and was further separated by preparative HPLC (Luna C18 100A, Phenomenex, 5 µ, 21.20 × 250 mm, gradient H_2_O:MeCN (50:50 v/v) to 20:80 v/v, in 30 min, 8 mL/min), to yield compounds **6** (46.5 mg) at 28 min and **7** (23.3 mg) at 19 min.

**1-(2,4-dihydroxy-5-methylphenyl)ethan-1-one (1):** Dark-brown oil; IR (neat) *ʋ*_max_, 3314 (br), 2971, 2853, 1652, 1406, 1038 cm^−1^; UV/Vis (MeOH) *λ*_max_ 194, 210, 232, 265, 360 nm. HRESIMS: *m*/*z* 165.0552 [M − H]^−^ (calcd for C_9_H_9_O_3_, 165.0552).

**(1’Z)-2-(1’,5’-dimethylhexa-1’,4’-dieny1)-5-methylbenzene-1,4-diol (2):** Dark-brown oil; IR (neat) *ʋ*_max_ 3413 (br), 2970, 2913, 1416, 1187 cm^−1^; UV/Vis (MeOH) *λ*_max_ 229, 299 nm. HRESIMS: *m*/*z* 231.1496 [M − H]^−^ (calcd for C_15_H_19_O_2_, 231.1385).

**6-(3-hydroxy-6-methyl-1,5-heptadien-2-yl)-3-methylbenzene-1,4-diol (3):** Yellow amorphous solid; (α)${}_{D}^{25}$ +0.72 (*c* 0.484, CH_3_OH); IR (MeOH) *ʋ*_max_ 3314 (br), 2943, 2831, 1033 cm^−1^; UV/Vis (CH_3_OH) *λ*_max_ 195, 299 nm. ^1^H-NMR (400 MHz, CDCl_3_) and ^13^C-NMR (100 MHz, CDCl_3_) see Table 1; HRESIMS: m/z 247.1344 [M − H]^−^ (calcd for C_15_H_19_O_3_, 247.1334), *m*/*z* 149.0575 (M − C_6_H_11_O)^−^ (calcd for C_9_H_9_O_2_, 149.0602).

**4-hydroxy-3,7-dimethyl-7-(3-methylbut-2-en-1-yl)benzofuran-15-one (4):** Yellow amorphous solid; (α)${}_{D}^{25}$ +2.2 (*c* 0.115, MeOH); IR (MeOH) *ʋ*_max_ 3313 (br), 2944, 2832, 1656, 1451, 1035 cm^−1^; UV/Vis (MeOH) *λ*_max_ 196, 294 nm. ^1^H-NMR (400 MHz, CDCl_3_) and ^13^C-NMR (100 MHz, CDCl_3_) see Table 1; HRESIMS: *m*/*z* 245.1126 [M − H]^−^ (calcd for C_15_H_17_O_3_, 245.1177).

**1,8-epoxy-1(6),2,4,7,10-bisaborapentaen-4-ol (5):** Yellow amorphous solid; IR (neat) *ʋ*_max_ 3266 (br), 2915, 1437, 1168, 805, 434 cm^−1^; UV/Vis (MeOH) *λ*_max_ 203, 257, 297 nm. HRESIMS: *m*/*z* 229.1234 [M − H]^−^ (calcd for C_15_H_17_O_2_, 229.1229).

**6-(2-methoxy-5-methylhept-4-en-2-yl)-3-methylbenzene-1,4-diol (6):** Dark-brown oil; (α)${}_{D}^{25}$ +5.0 (*c* 0.0337, CH_3_OH); IR (MeOH) *ʋ*_max_ 3339 (br), 2926, 1453, 1374, 1183, 1051 cm^−1^; UV/Vis (CH_3_OH) *λ*_max_ 196, 297 nm. ^1^H-NMR (400 MHz, CDCl_3_) and ^13^C-NMR (100 MHz, CDCl_3_) see Table 2; HRESIMS: *m*/*z* 263.1610 [M − H]^−^ (calcd for C_16_H_23_O_3_, 263.1647).

**9-(3,3-dimethyloxiran-2-yl)-1,7-dimethyl-7-chromen-4-ol (7):** Green crystals; (α)${}_{D}^{25}$ −10.4 (*c* 0.0322, CH_3_OH); IR (neat) *ʋ*_max_ 3388 (br), 2926, 1412, 1178, 994, 829, 597 cm^−1^; UV/Vis (CH_3_OH) *λ*_max_ 194, 217, 330 nm. ^1^H-NMR (400 MHz, CDCl_3_) and ^13^C-NMR (100 MHz, CDCl_3_) see Table 2; HRESIMS: *m*/*z* 245.1126 [M − H]^−^ (calcd for C_15_H_17_O_3_, 245.1177).

### 3.4. Biological Activities

#### 3.4.1. Cytotoxicity

The cytotoxic activity of compounds **1**–**7** was tested against A-549 human lung carcinoma cells, MDA-MB-231 human breast adenocarcinoma cells, HT-29 human colorectal carcinoma cells, and PSN1 human pancreatic adenocarcinoma cells. The four cell lines were provided by the American Type Culture Collection (ATCC): A549 from ATCC CCL-185, MDA-MB-231 from ATCC HTB-26, HT-29 from ATCC HTB-38 and PSN-1 from ATCC CRM-CRL-3211. The concentration giving half maximum inhibitory concentration (IC_50_) was calculated according to the procedure described in the literature [32]. Cell survival was estimated using the National Cancer Institute (NCI) algorithm [33]. Dose-response parameters were determined at three different concentrations of each one of the compounds.

#### 3.4.2. Zebrafish Nile Red Fat Metabolism Assay

The lipid reducing activity of the compounds was analyzed using the zebrafish Nile red fat metabolism assay as previously described [16,25]. Approval by an ethics committee was not necessary for the presented work since the procedures used are not considered animal experimentation according to EC Directive 86/609/EEC for animal experiments. In brief, zebrafish embryos were raised from 1 DPF (days post fertilization) in egg water (60 µg/mL marine sea salt dissolved in distilled H_2_O) with 200 µM PTU (1-phenyl-2-thiourea) to inhibit pigmentation. From 3 DPF to 5 DPF, zebrafish larvae were exposed to compounds at a final concentration of 10 µM with the daily renewal of water and compounds in a 48 well plate with a density of 6–8 larvae/well (*n* = 6–8). A solvent control (0.1% DMSO) and positive control (REV, resveratrol, final concentration of 50 µM) were included in the assay. Lipids were stained with Nile red overnight at the final concentration of 10 ng/mL. For imaging, the larvae were anesthetized with tricaine (MS-222, 0.03%) for 5 minutes and fluorescence analyzed with a fluorescence microscope (Olympus BX43, Hamburg, Germany). Fluorescence intensity was quantified in individual zebrafish larvae by ImageJ [34]. Effective concentrations 50% (EC_50_) values were determined in further assays by dose-response curves by using a 1:2 v/v dilution series from 20 µM to 312.5 nM (final concentrations) in 7 dilution steps.

## 4. Conclusions

This work represents the first isolation and structural elucidation of novel compounds **3**, **4**, **6**, and **7**. It is also the first report of the isolation of compounds **1**, **2**, and **5** from marine sources. Besides being a known and wide-spread class of compounds, the structures of the new compounds isolated present unique structural features. The isolation of these novel compounds, as well as related analogs previously found in marine-derived organisms, raises the question of who is the real metabolite producer, the host or the associated-microorganisms. Further studies are needed in order to answer that question. All of the isolated compounds except for **1** and **6**, showed significant lipid-reducing activity when tested in the zebrafish Nile red fat metabolism assay, but no general toxicity, reinforcing their biotechnological potential. More studies are needed in order to relate the bioactivity with structural features.

## Figures and Tables

**Table 1:** ^1^H and ^13^C-NMR (400 and 100 MHz, respectively) for compounds **3** and **4**. The experiments were performed in CDCl_3_.

Position	Compound 3		Compound 4	
	δ_C_, Type	δ_H_, mult (*J* in Hz)	δ_C_, Type	δ_H_, mult (*J* in Hz)
1	147.5, C		146.4, C	
2	118.8, CH	6.68, s	112.5, CH	6.87, s
3	125.4, C		123.9, C	
4	147.0, C		150.4, C	
5	117.2, CH	6.50, s	110.0, CH	6.64, s
6	124.2, C		130.4, C	
7	148.0, C		48.1, C	
8	76.7, CH	4.40, dd (8.6, 5.4)	37.4, CH_2_	2.42, dd (14.1, 7.9) 2.58, dd (14.1, 8.3)
9	34.3, CH_2_	α 2.30, m β 2.15, m	117.2, CH	4.85, dddd (9.7, 5.5, 2.8, 1.4)
10	118.8, CH	5.06, m	136.5, C	
11	136.9, C		18.0, CH_3_	1.56, s
12	26.1, CH_3_	1.71, s	25.8, CH_3_	1.60, s
13	18.1, CH_3_	1.53, s	23.5, CH_3_	1.44, s
14	15.8, CH_3_	2.20, s	16.2, CH_3_	2.26, s
15	120.3, CH_2_	α 5.43, d (1.3) β 5.24, d (1.6)	180.8, C	
OH-1		8.02, br s		
OH-4		4.48, br s		4.65, br s
OH-8		3.27, br s		

**Table 2:** ^1^H and ^13^C-NMR (400 and 100 MHz, respectively) for compounds **6** and **7**. Experiments with compound **6** were done in CD_3_OD and with compound **7** in CDCl_3_.

Position	Compound 6		Compound 7	
	δ_C_, Type	δ_H_, mult (*J* in Hz)	δ_C_, Type	δ_H_, mult (*J* in Hz)
1	149.5, C		146.7, C	
2	118.9, CH	6.63, s	116.9, CH	6.70, s
3	124.5, C		125.1, C	
4	146.7, C		148.1, C	
5	114.2, CH	6.48, s	110.3, CH	6.62, s
6	126.0, C		132.1, C	
7	82.4, C		122.0, C	
8	39.8, CH_2_	1.84, m	118.7, C	5.36, dd (3.8, 1.5)
9	22.8, CH_2_	α 2.00, m β 1.89, m	75.6, CH	4.51, ddt (8.3, 3.9, 1.5)
10	123.9, CH	5.04, t (6.6, 6.5)	63.8, CH	3.06, d (8.3)
11	132.0, C		57.7, C	
12	17.7, CH_3_	1.51, m	25.1, CH_3_	1.33, s
13	25.8, CH_3_	1.65, s	19.4, CH_3_	1.35, s
14	15.6, CH_3_	2.18, s	15.9, CH_3_	2.19, s
15	22.4, CH_3_	1.55, s	18.3, CH_3_	2.01, t (1.5)
16	50.5, OCH_3_	3.21, s		
OH-1		8.28, br s		
OH-4		8.28, br s		3.49, br s

## Supplementary Materials

The following are available online at https://www.mdpi.com/1660-3397/17/6/375/s1. Figure S1: Picture of the fresh sponge; Figures S2–S44: HRESIMS and NMR spectra of compounds **1**–**7**; Figure S45: MS fragmentation pattern of compound **7**.
