# Supplementary material for: New Aromatic Bisabolane Derivatives with Lipid-Reducing Activity from the Marine Sponge Myrmekioderma sp
Source: Mar Drugs. 2019 Jun 22;17(6):375. doi: 10.3390/md17060375 (PMC6627430; doi:10.3390/md17060375)
Supplement: Supplementary file 1 [file marinedrugs-17-00375-s001.pdf]

New aromatic bisabolane derivatives with anti-obesity activity

Margarida Costa [1*], Laura Coello [2], Ralph Urbatzka [3], Marta Pérez [2], Margrét Thorsteinsdóttir[1]

[1] Faculty of Pharmaceutical Sciences, University of Iceland, Hofsvallagata 53, 107 Reykjavik, Iceland; costa.anamarg@gmail.com
[2] Research & Development Department, PharmaMar S.A., Pol. Ind. La Mina Norte, Avda. de los Reyes 1, 28770 Colmenar Viejo (Madrid), Spain
[3] Interdisciplinary Centre of Marine and Environmental Research (CIIMAR/CIMAR), University of Porto, Avenida General Norton de Matos, s/n, 4450-208 Matosinhos, Portugal
* Correspondence: margreth@hi.is; Tel.: +354-895-4761

Table of content **Pages**

Figure S1 – Fresh sponge used in this study. 2

Figures S2-S3 - 1D NMR spectra for **Compound 1** in $CDCl_3$ 3-4

Figure S4 - HRESIMS spectrum for **Compound 1** 5

Figures S5-S7 - 1D NMR and *g*-COSY spectra for **Compound 2** in $CDCl_3$ 5-8

Figure S8 - HRESIMS spectrum for **Compound 2** 9

Figures S9-S15 - 1D and 2D NMR spectra for **Compound 3** in $CDCl_3$ 10-16

Figure S16 - HRESIMS spectrum for **Compound 3** 17

Figures S17-S23 - 1D and 2D NMR spectra for **Compound 4** in $CDCl_3$ 18-24

Figure S24 - HRESIMS spectrum for **Compound 4** 25

Figures S25-S26 - 1D and spectra for **Compound 5** in $CDCl_3$ 26-27

Figure S27 - HRESIMS spectrum for **Compound 5** 28

Figures S28-S34 - 1D and 2D NMR spectra for **Compound 6** in CH_3OD 29-35

Figure S35 - HRESIMS spectrum for **Compound 6** 36

Figures S36-S43 - 1D and 2D NMR spectra for **Compound 7** in $CDCl_3$ 37-44

Figure S44 - HRESIMS spectrum for **Compound 7** 45

Figure S45 – MS fragmentation pattern for **Compound 7** 46

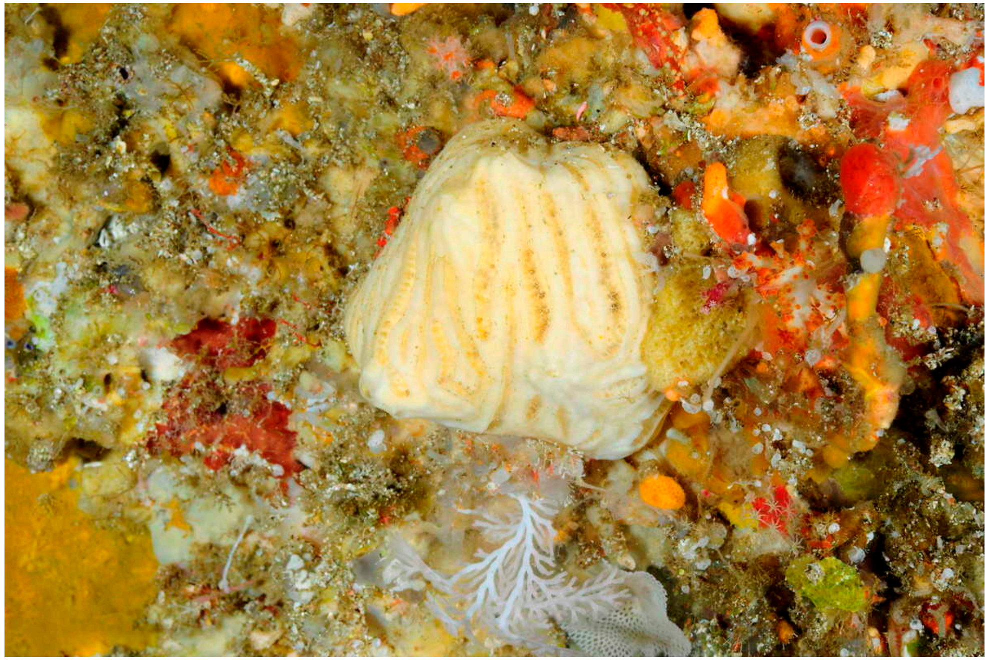

Figure S1 – Fresh sponge used for this study. The sample was morphologically identified as *Myrmekioderma* sp..

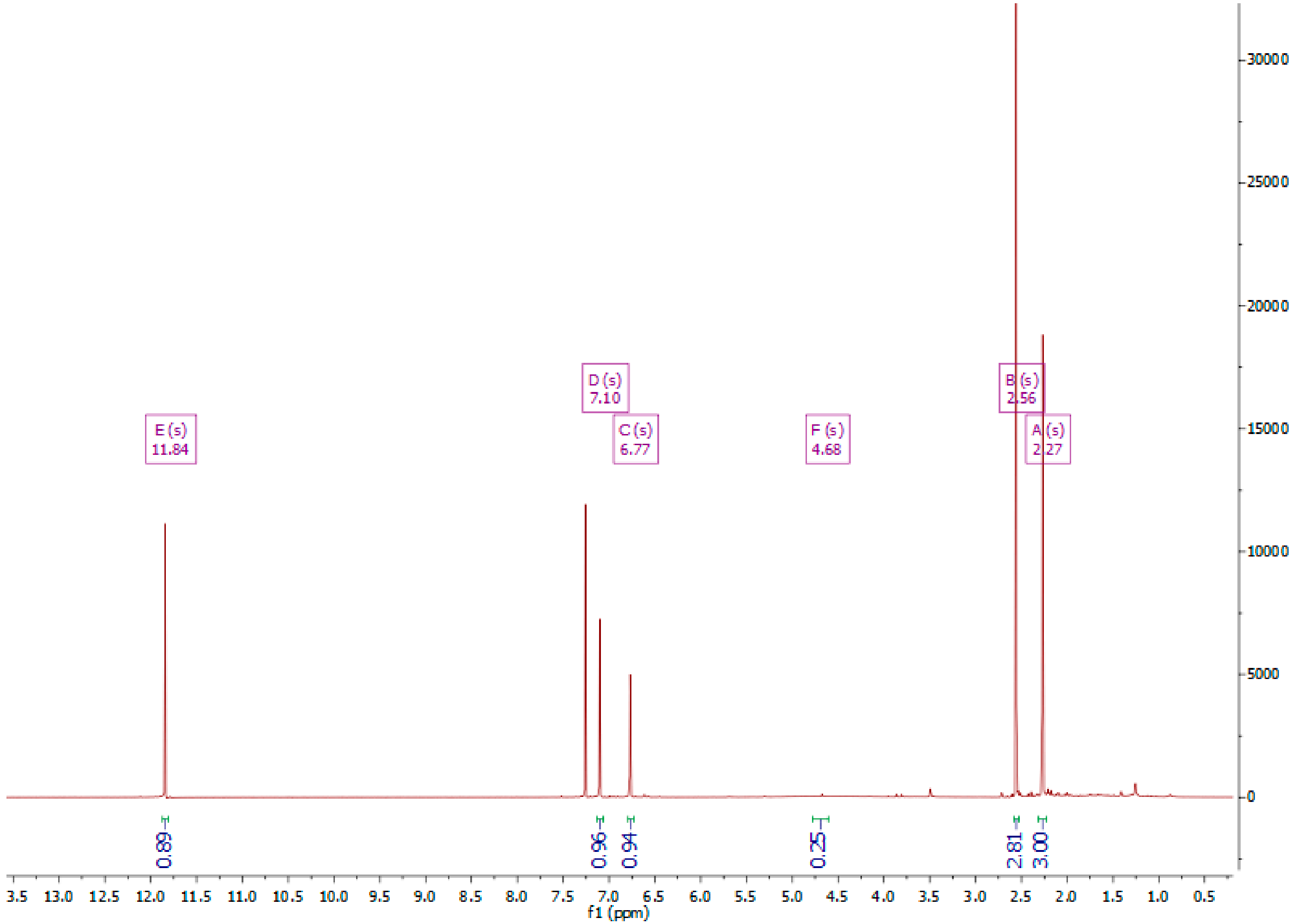

Figure S2 – ^{1}H-NMR spectrum for **Compound 1** (400 MHz, $CDCl_3$).

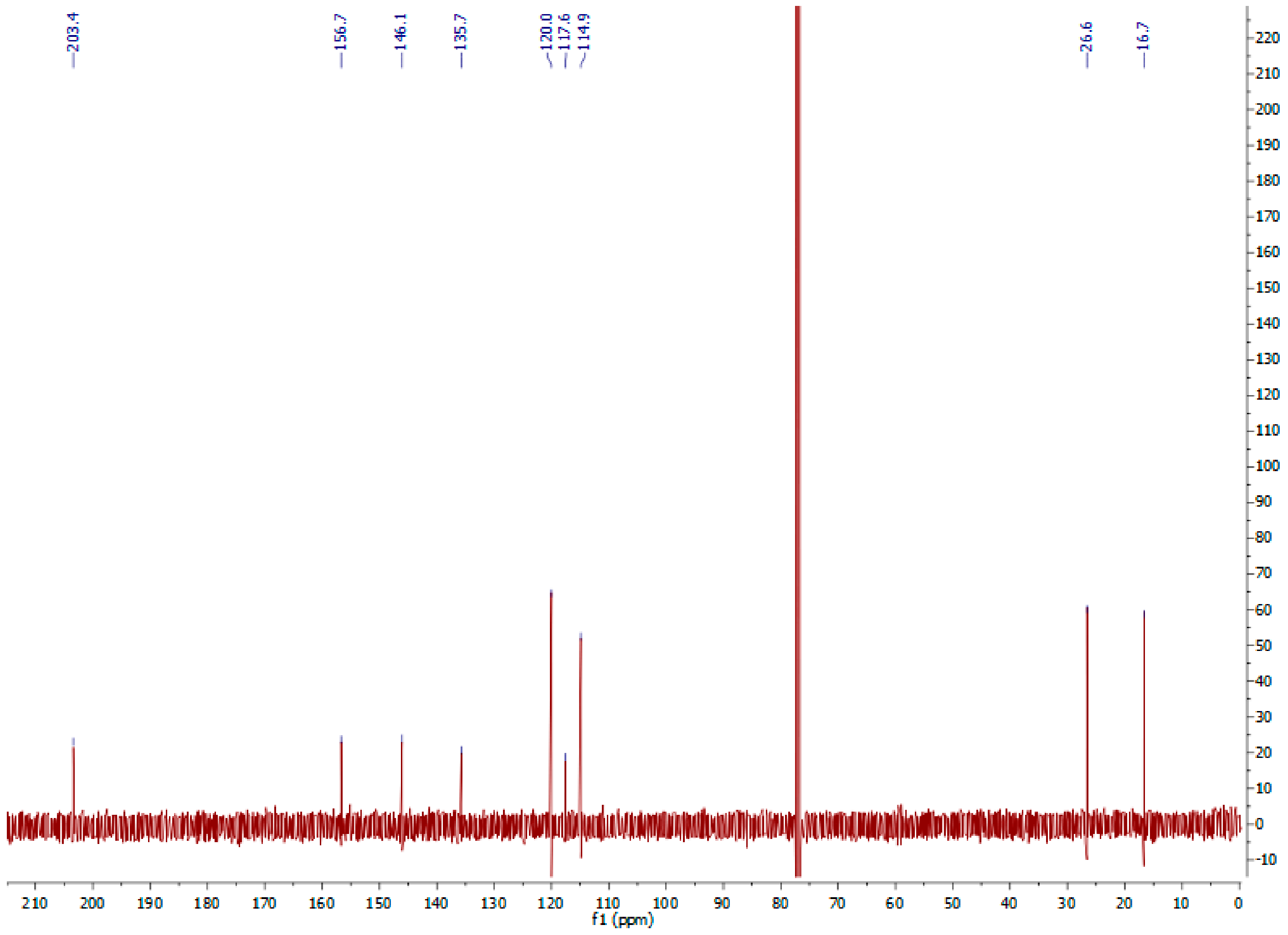

Figure S3 – ^{13}C-NMR spectrum for **Compound 1** (100 MHz, $CDCl_3$).

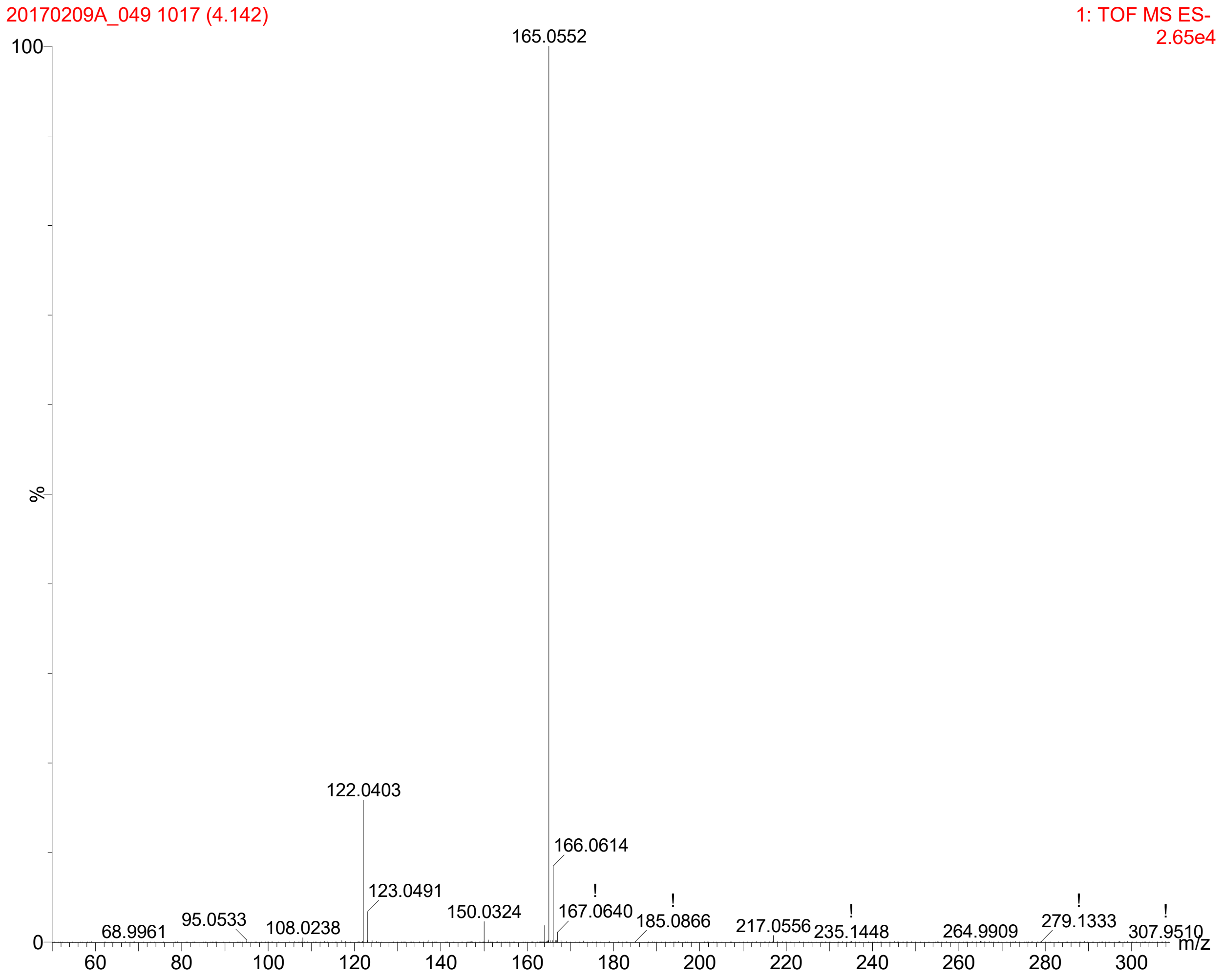

Figure S4 - HRESIMS spectrum for **Compound 1**.

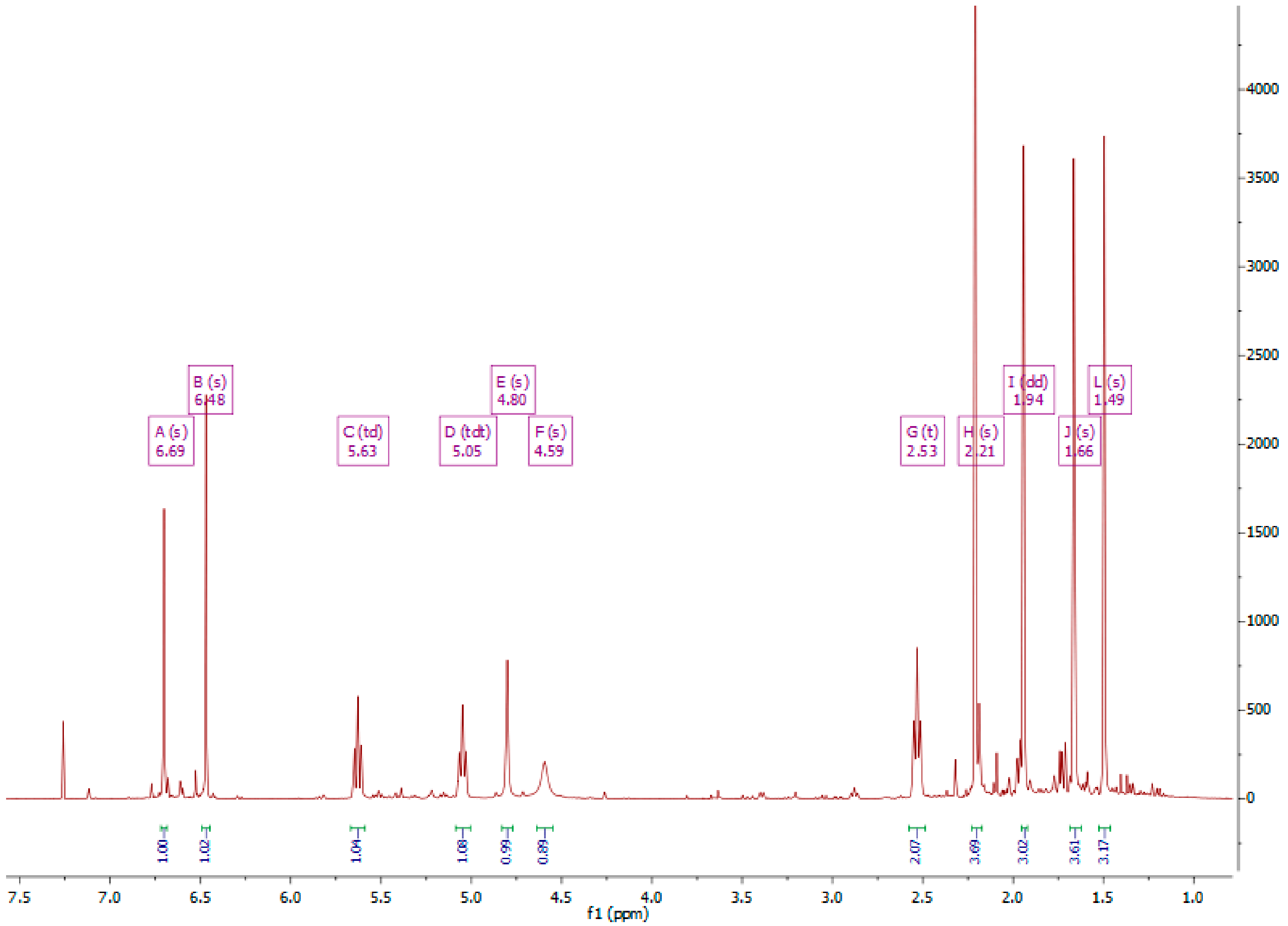

Figure S5 – ^{1}H-NMR spectrum for **Compound 2** (400 MHz, ($CDCl_3$).

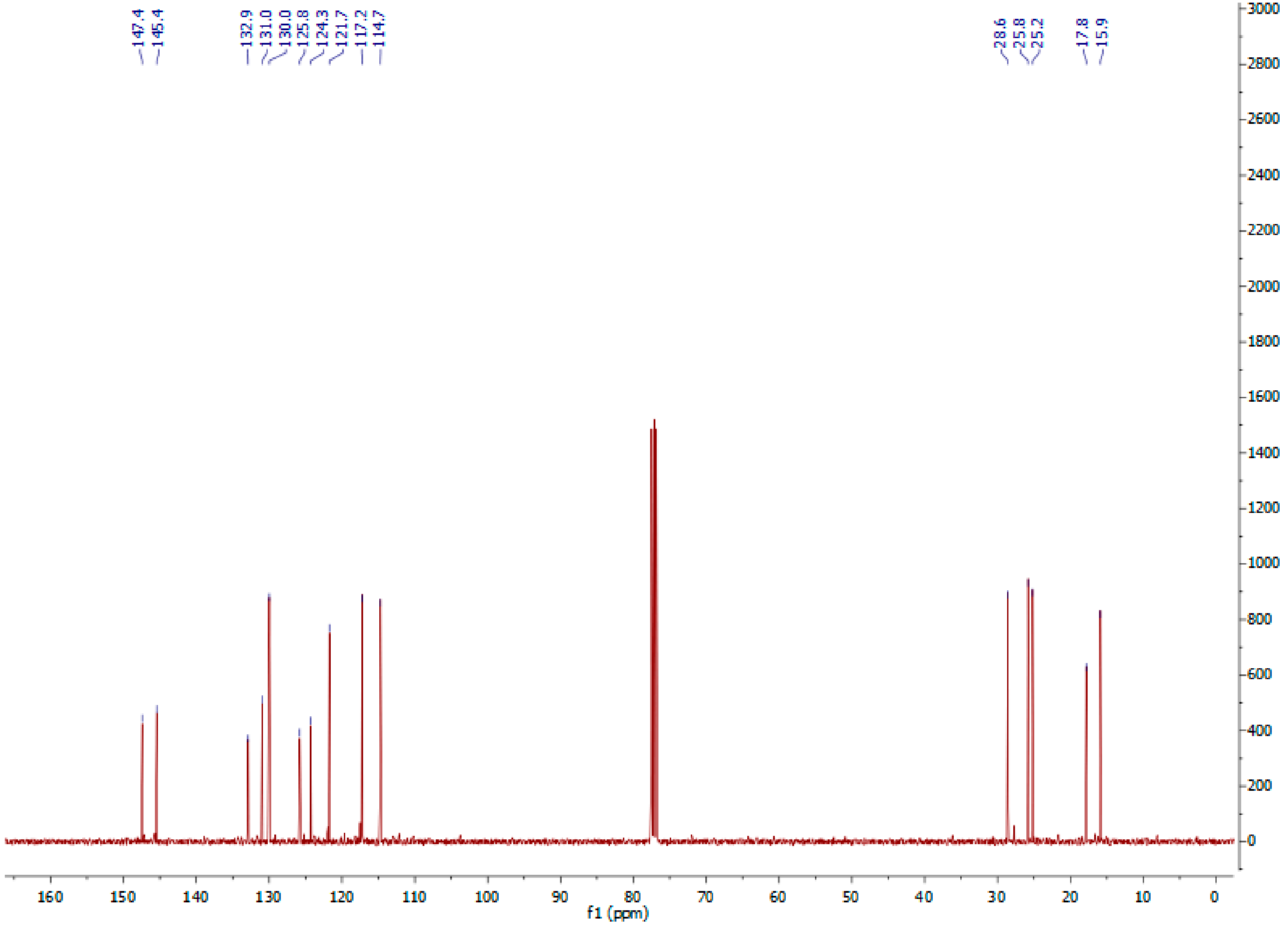

Figure S6 – ^{13}C-NMR spectrum for **Compound 2** (100 MHz, $CDCl_3$).

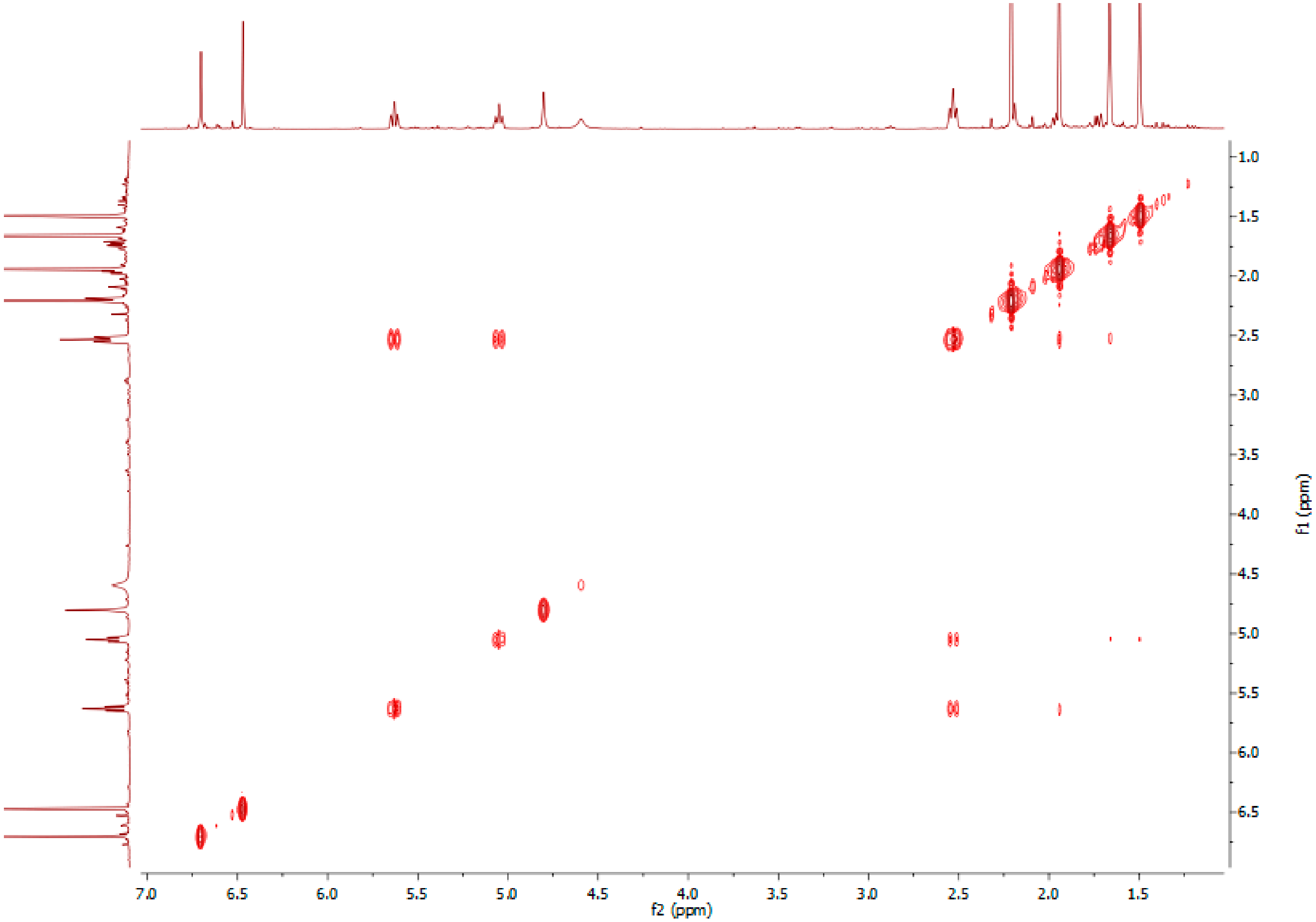

Figure S7 – *g*-COSY spectrum for **Compound 2** (100 MHz, $CDCl_3$).

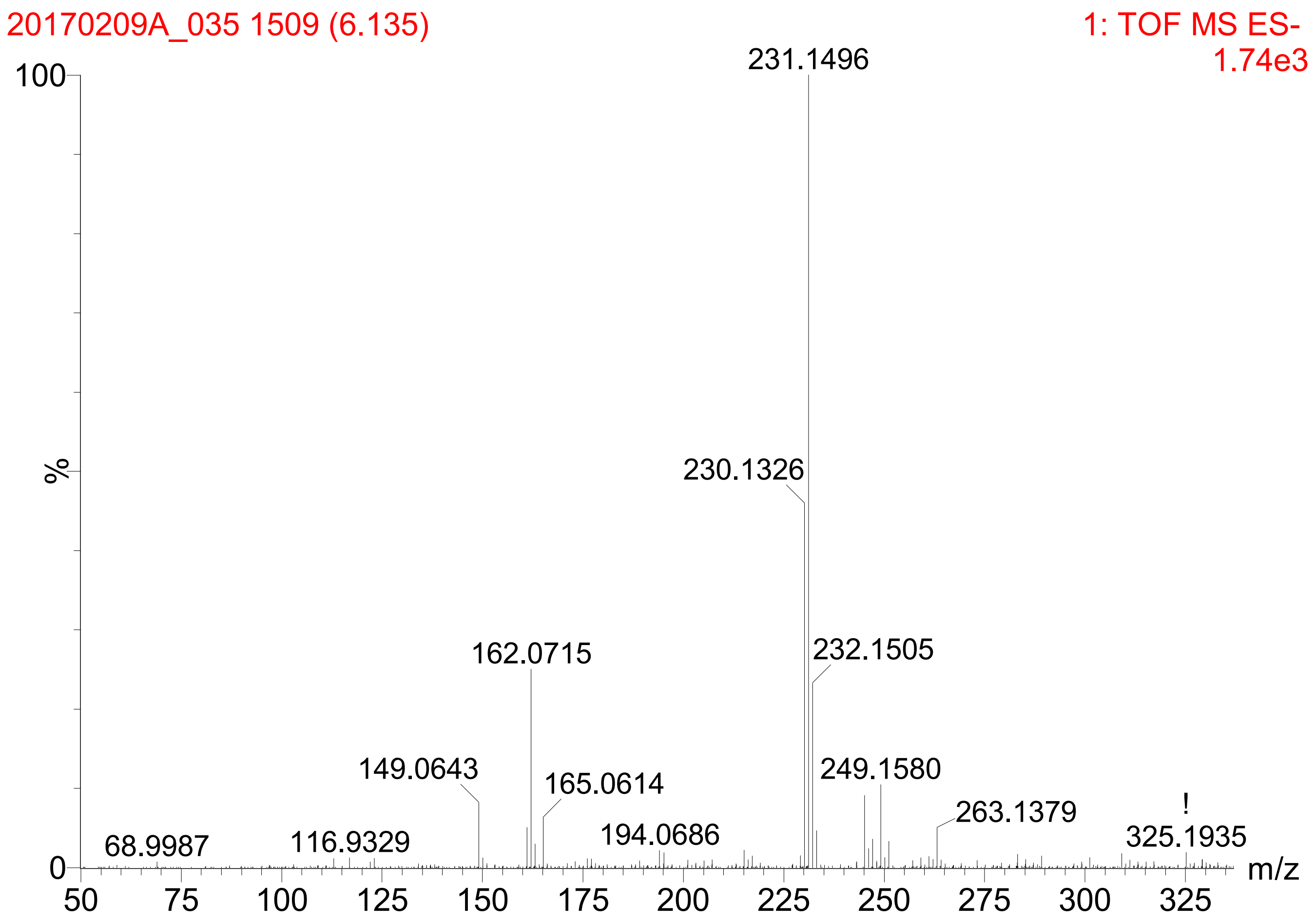

Figure S8 - HRESIMS spectrum for **Compound 2**.

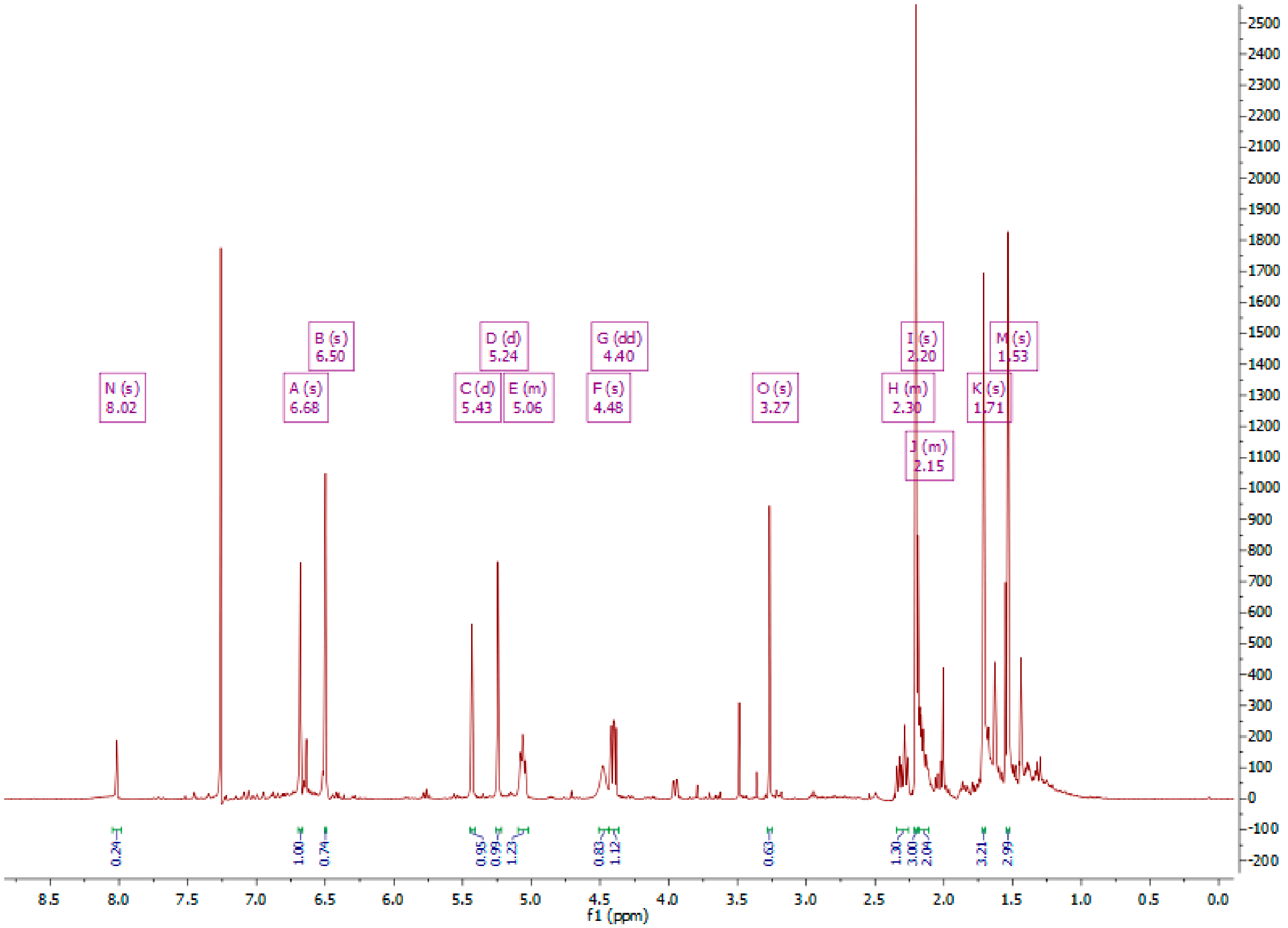

Figure S9 – ^{1}H-NMR spectrum for **Compound 3** (400 MHz, $CDCl_3$).

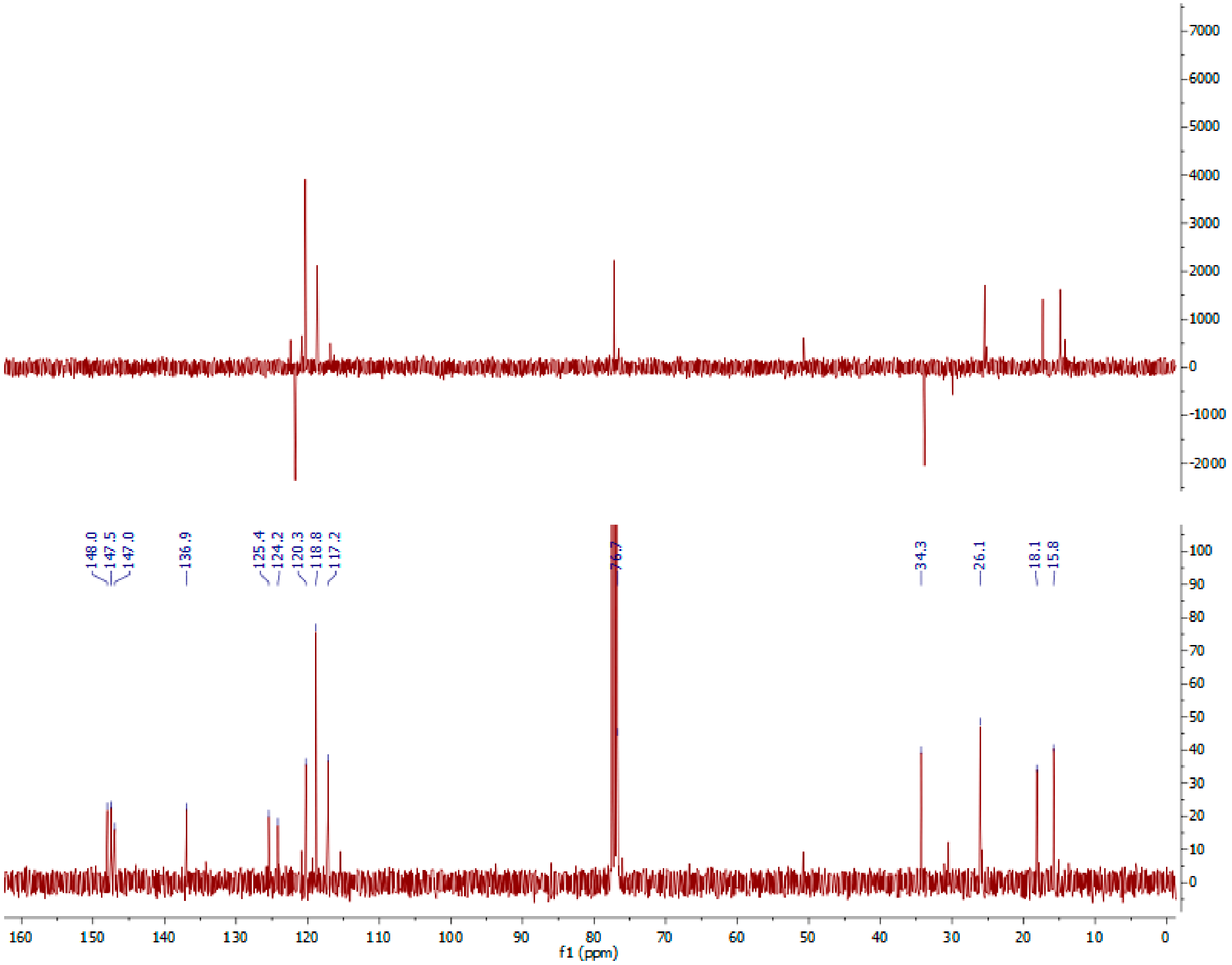

Figure S10 – DEPT and ^{13}C-NMR spectra for **Compound 3** (100 MHz, $CDCl_3$).

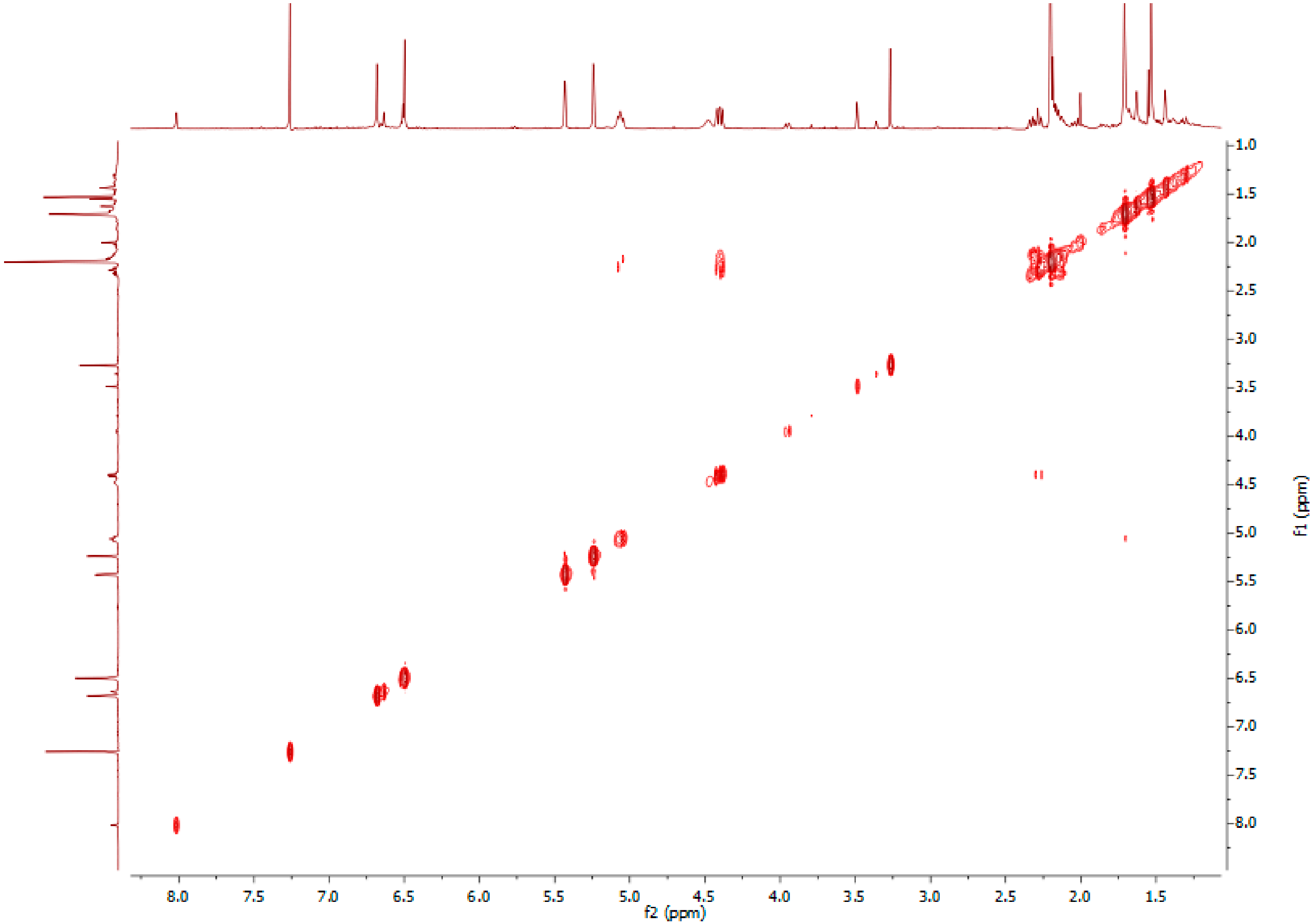

Figure S11 - *g*-COSY spectrum for **Compound 3** (400 MHz, $CDCl_3$).

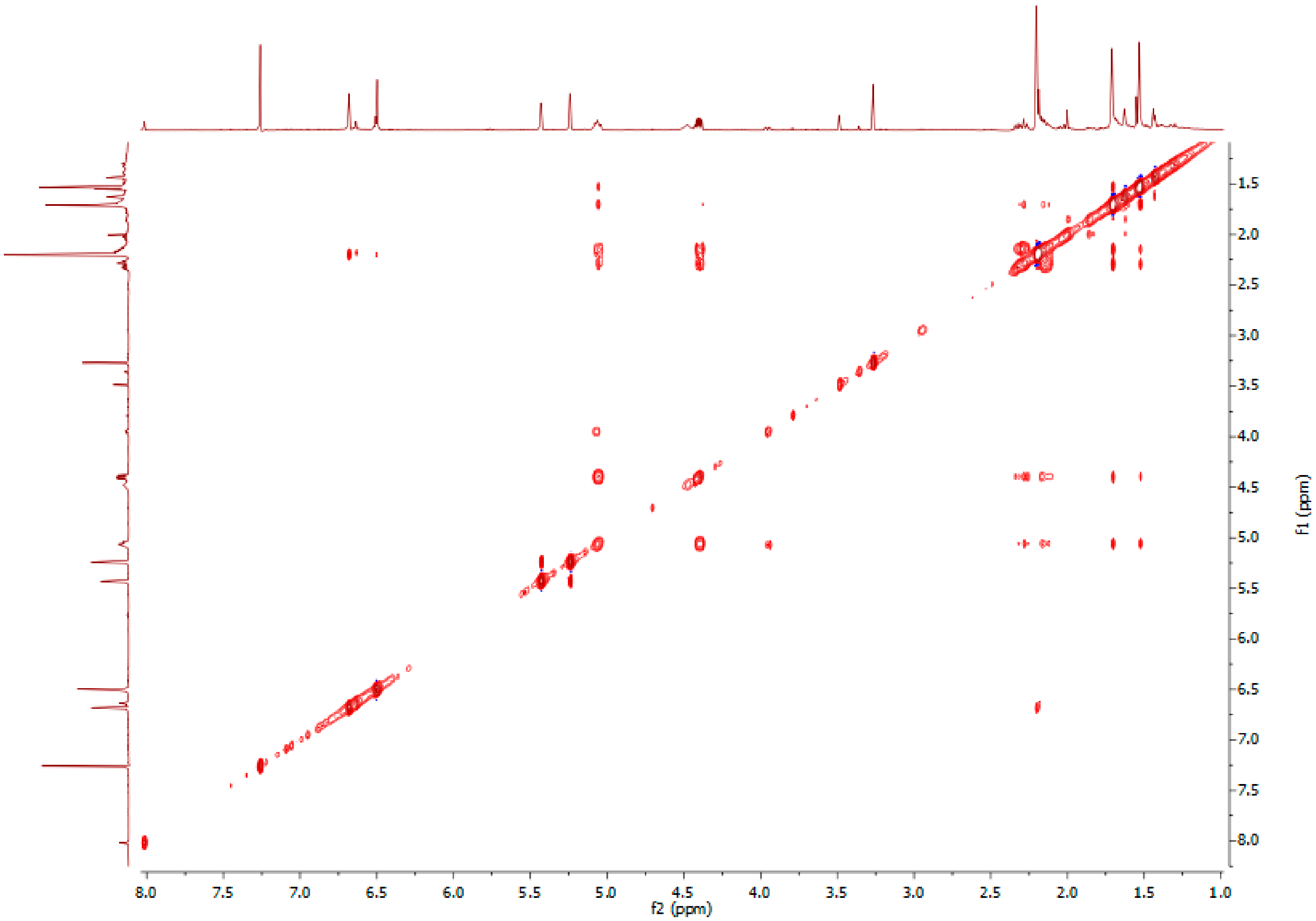

Figure S12 - TOCSY spectrum for **Compound 3** (400 MHz, $CDCl_3$).

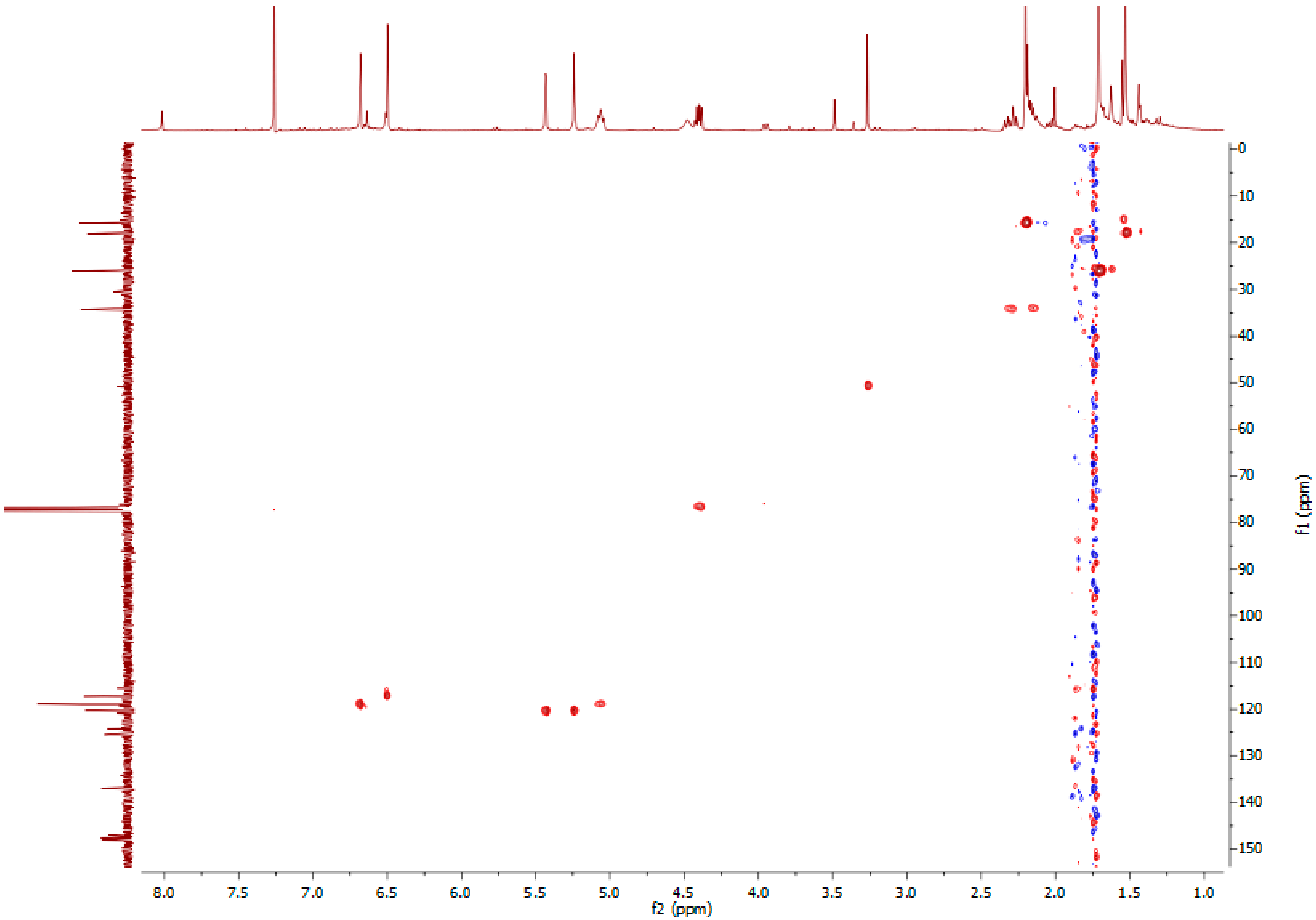

Figure S13 - *g*-HMQC spectrum for **Compound 3** (400 MHz, $CDCl_3$).

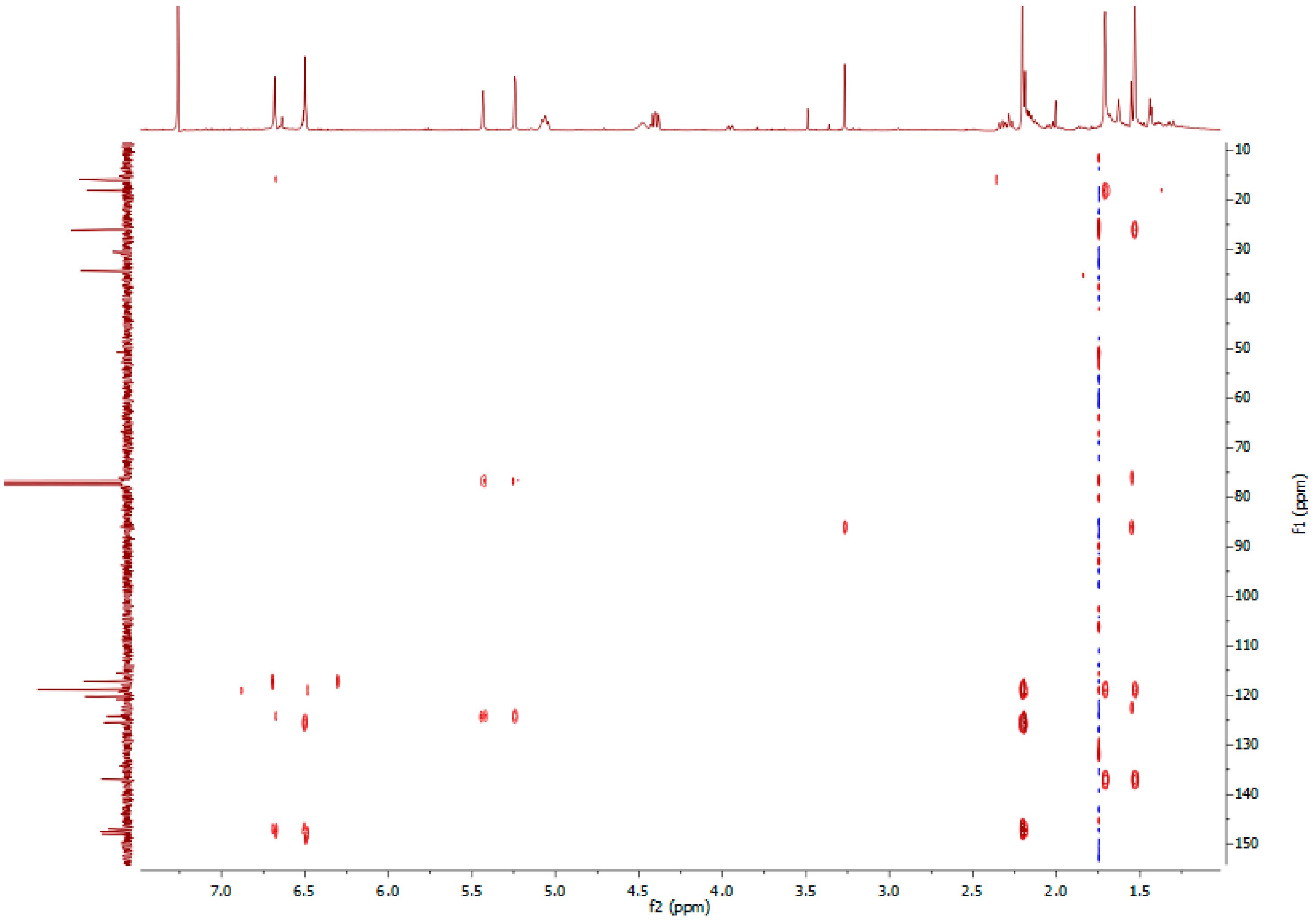

Figure S14 - *g*-HMBC spectrum for **Compound 3** (400 MHz, $CDCl_3$).

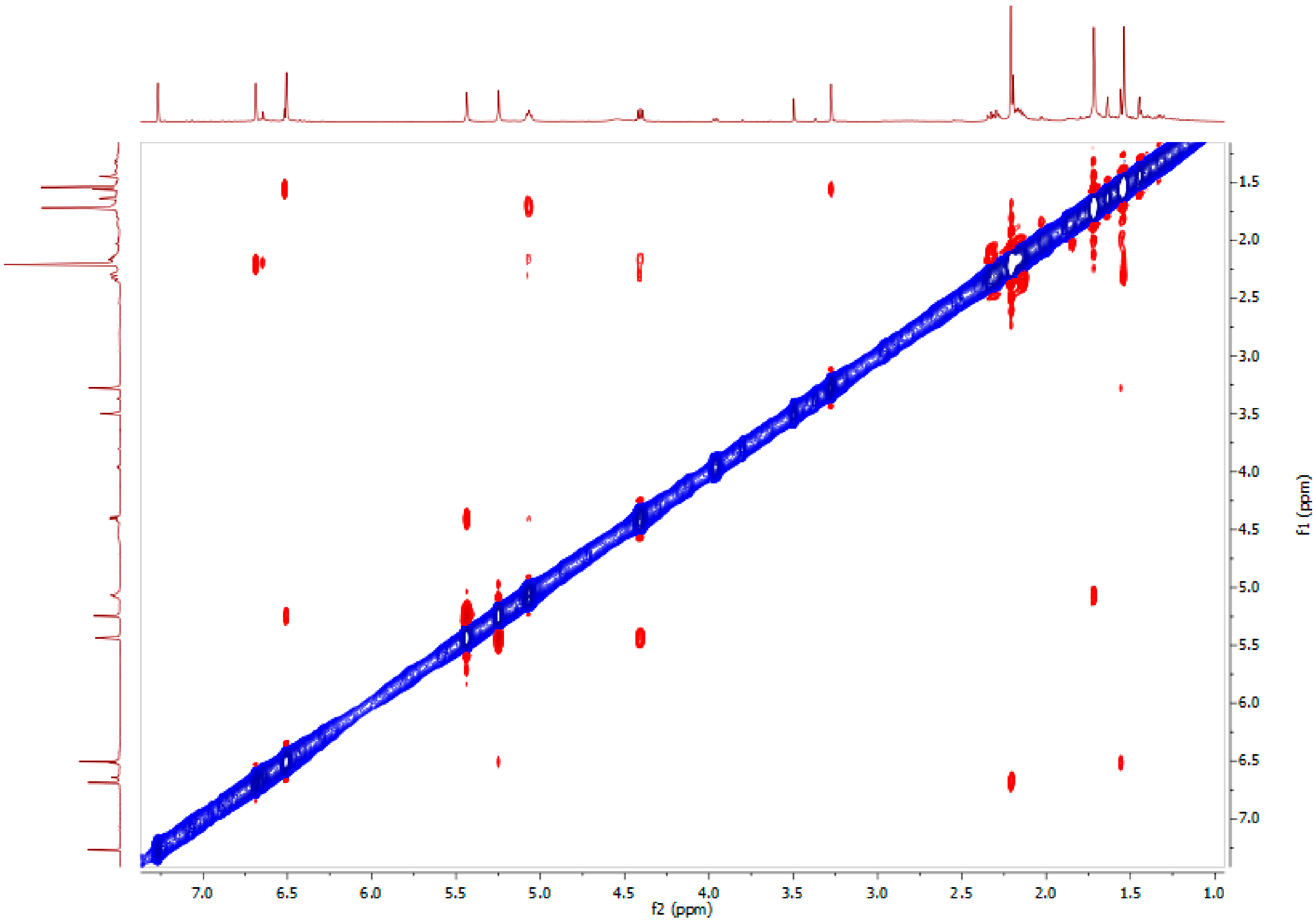

Figure S15 - NOESY spectrum for **Compound 3** (400 MHz, $CDCl_3$).

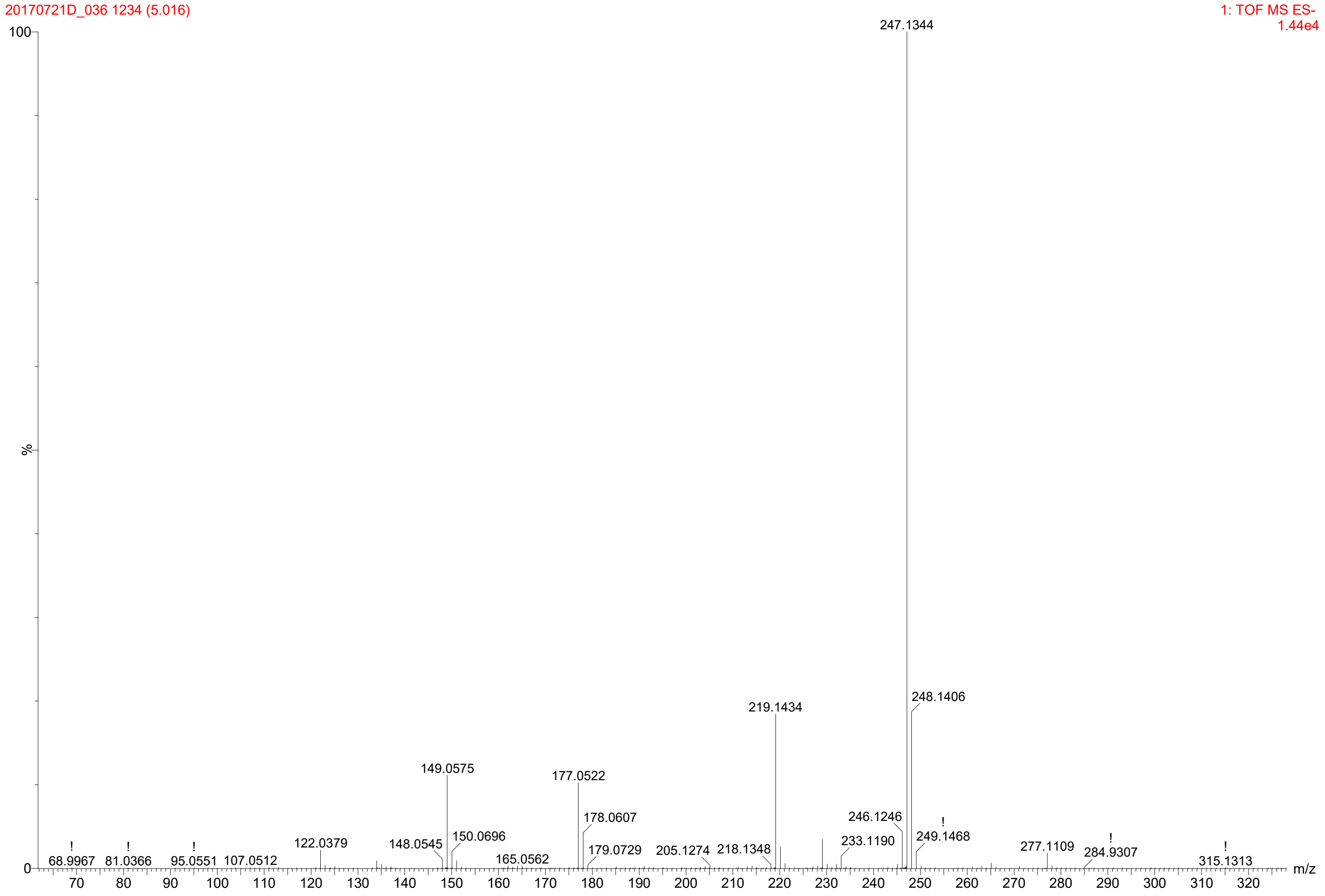

Figure S16 - (-)-HRESIMS spectrum for **Compound 3**.

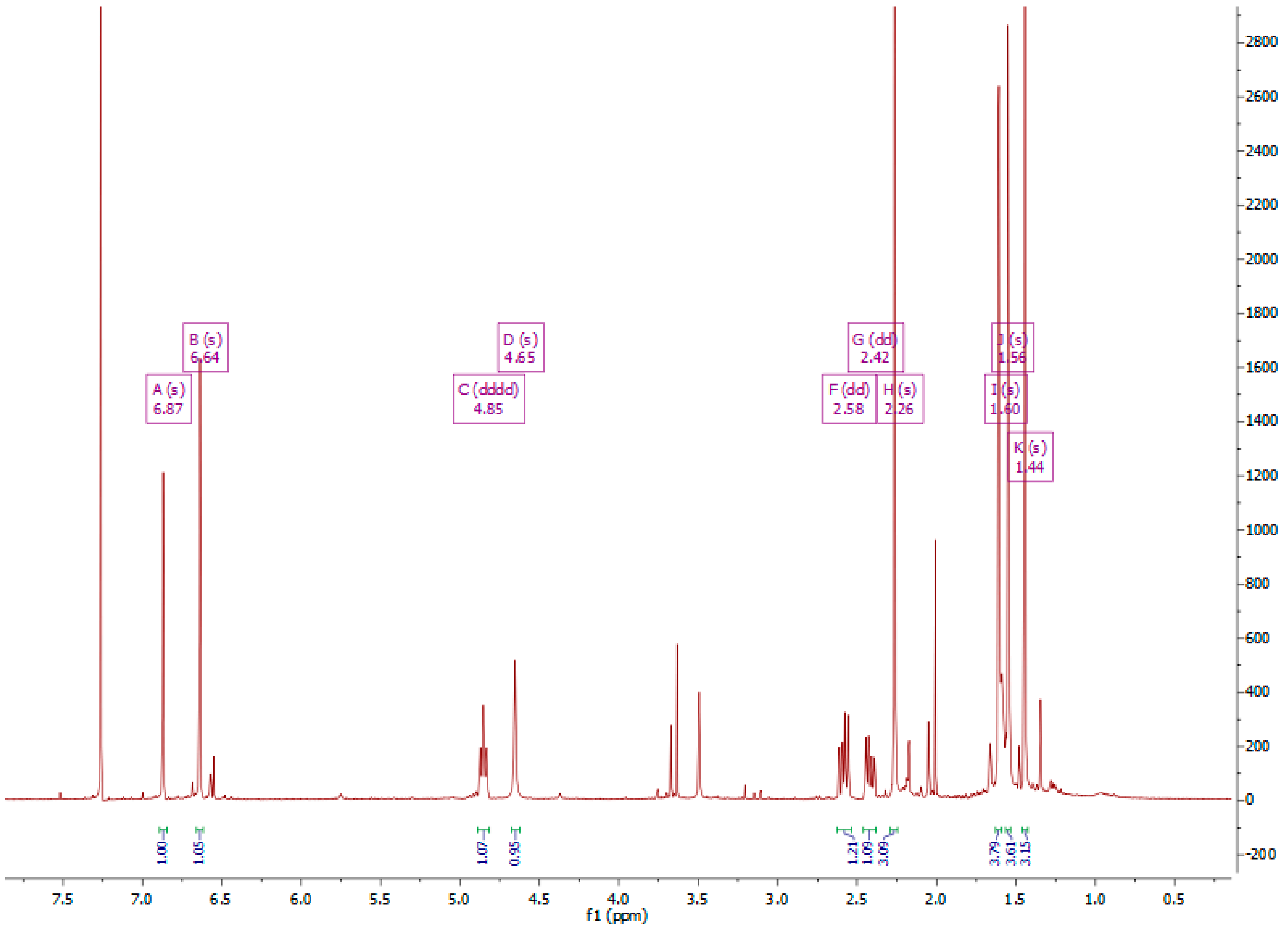

Figure S17 – ^{1}H-NMR spectrum for **Compound 4** (400 MHz, $CDCl_3$).

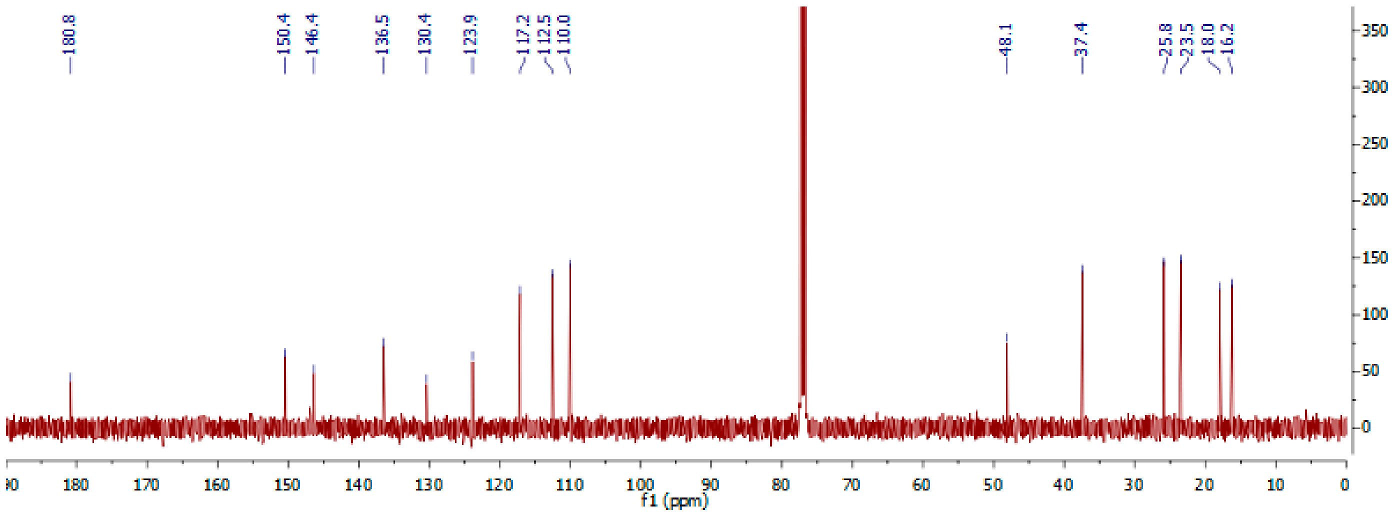

Figure S18 –^{13}C-NMR spectra for **Compound 4** (100 MHz, $CDCl_3$).

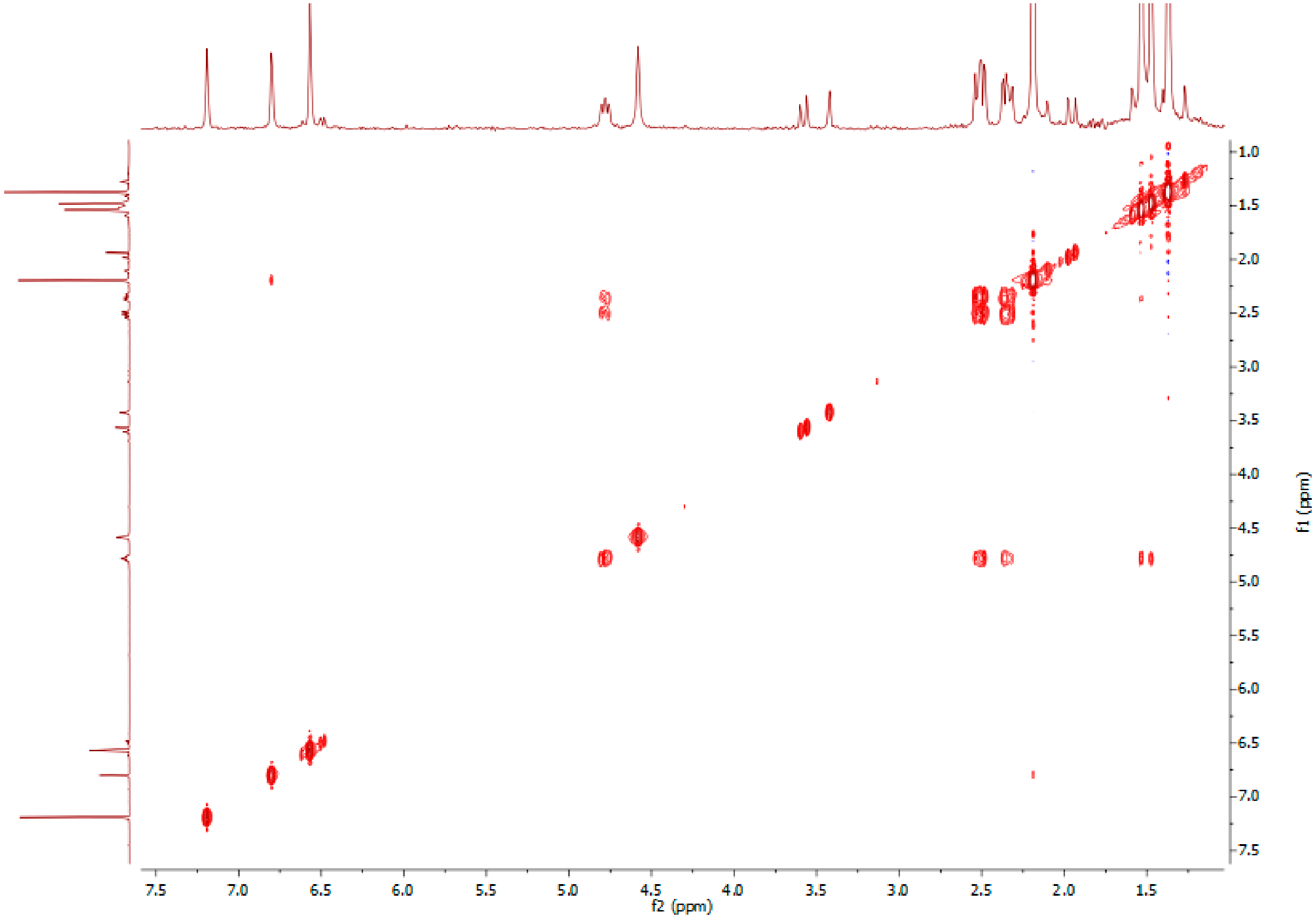

Figure S19 - *g*-COSY spectrum for **Compound 4** (400 MHz, $CDCl_3$).

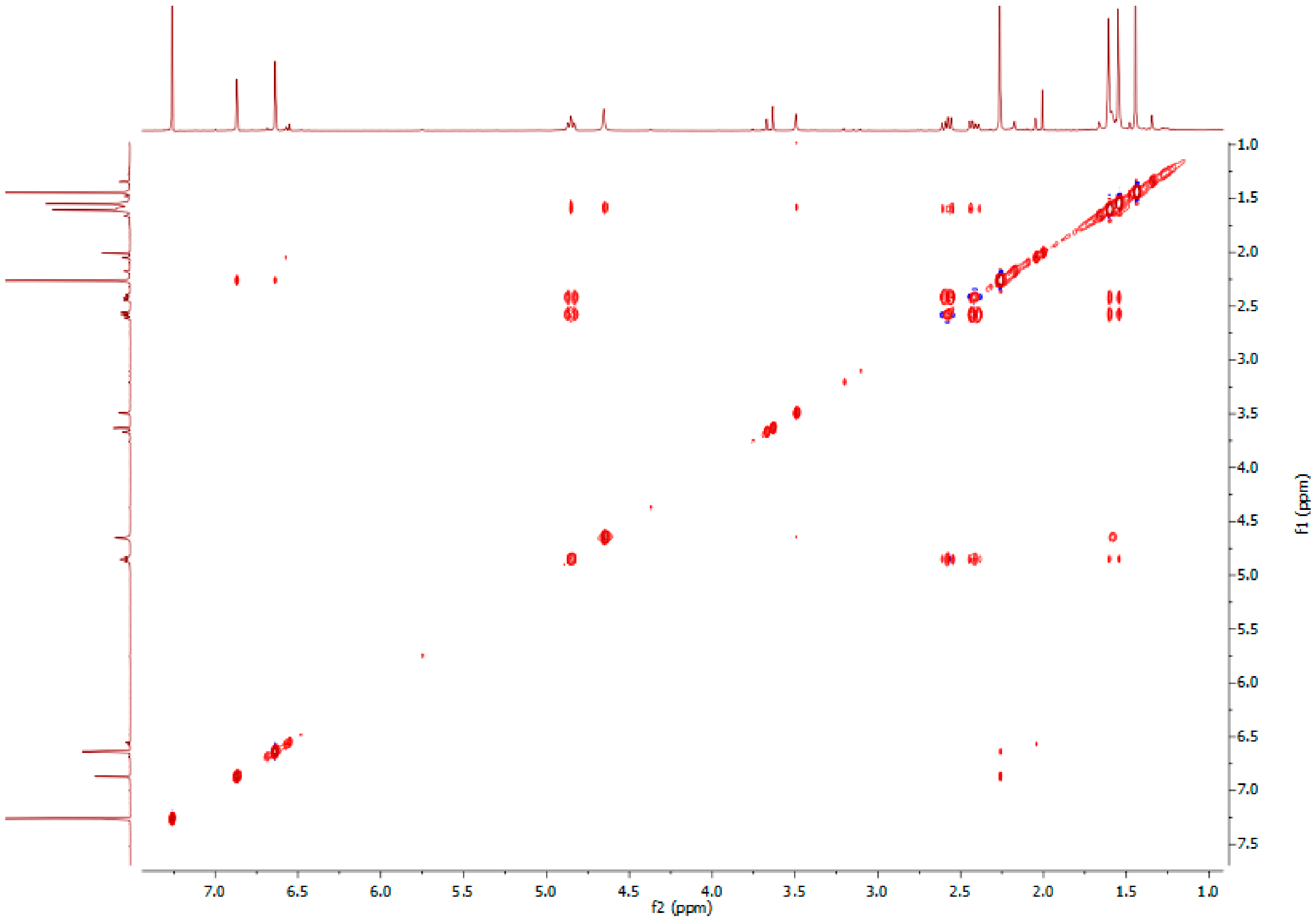

Figure S20 - TOCSY spectrum for **Compound 4** (400 MHz, $CDCl_3$).

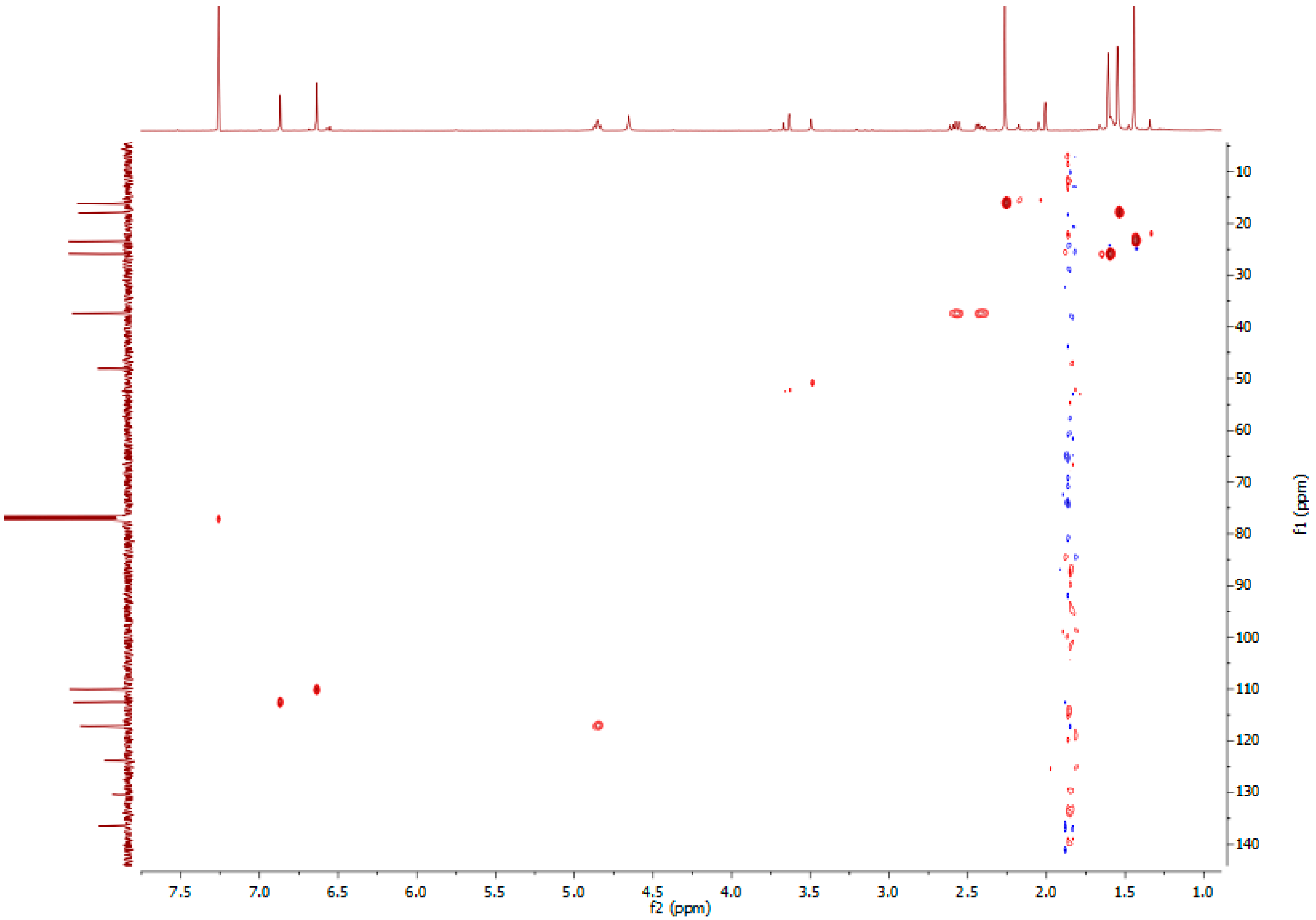

Figure S21 - *g*-HMQC spectrum for **Compound 4** (400 MHz, $CDCl_3$).

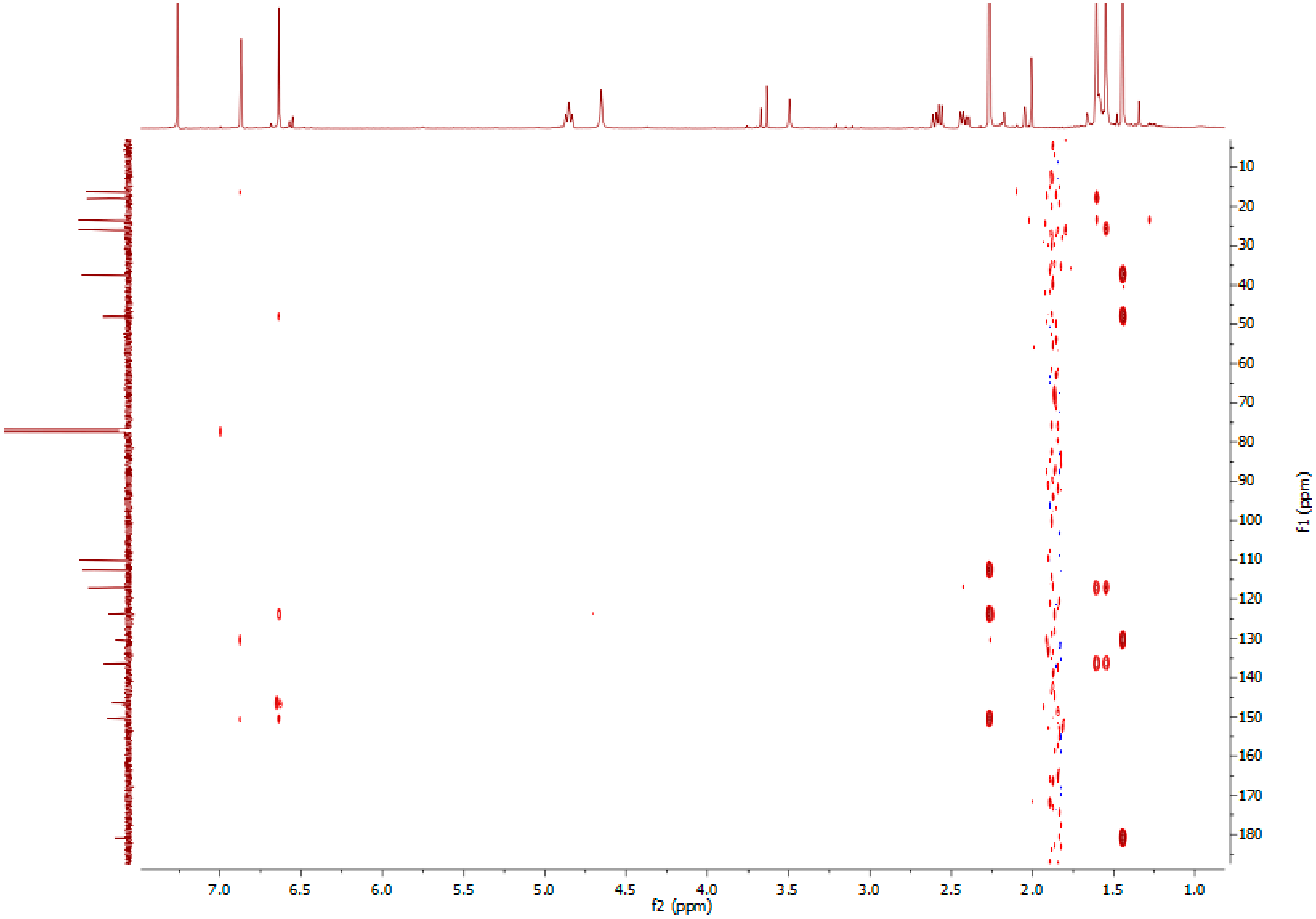

Figure S22 - *g*-HMBC spectrum for **Compound 4** (400 MHz, $CDCl_3$).

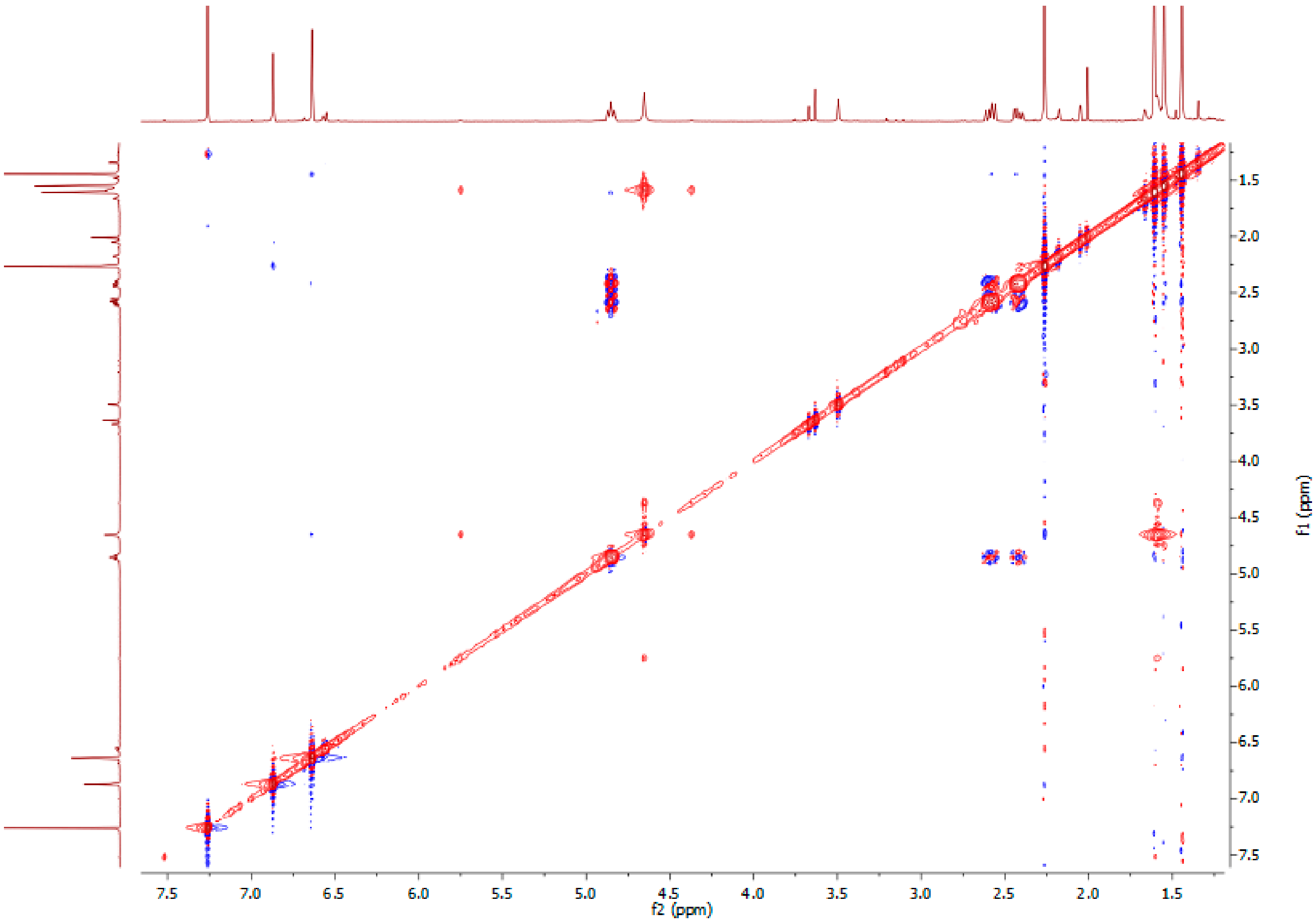

Figure S23 - NOESY spectrum for **Compound 4** (400 MHz, $CDCl_3$).

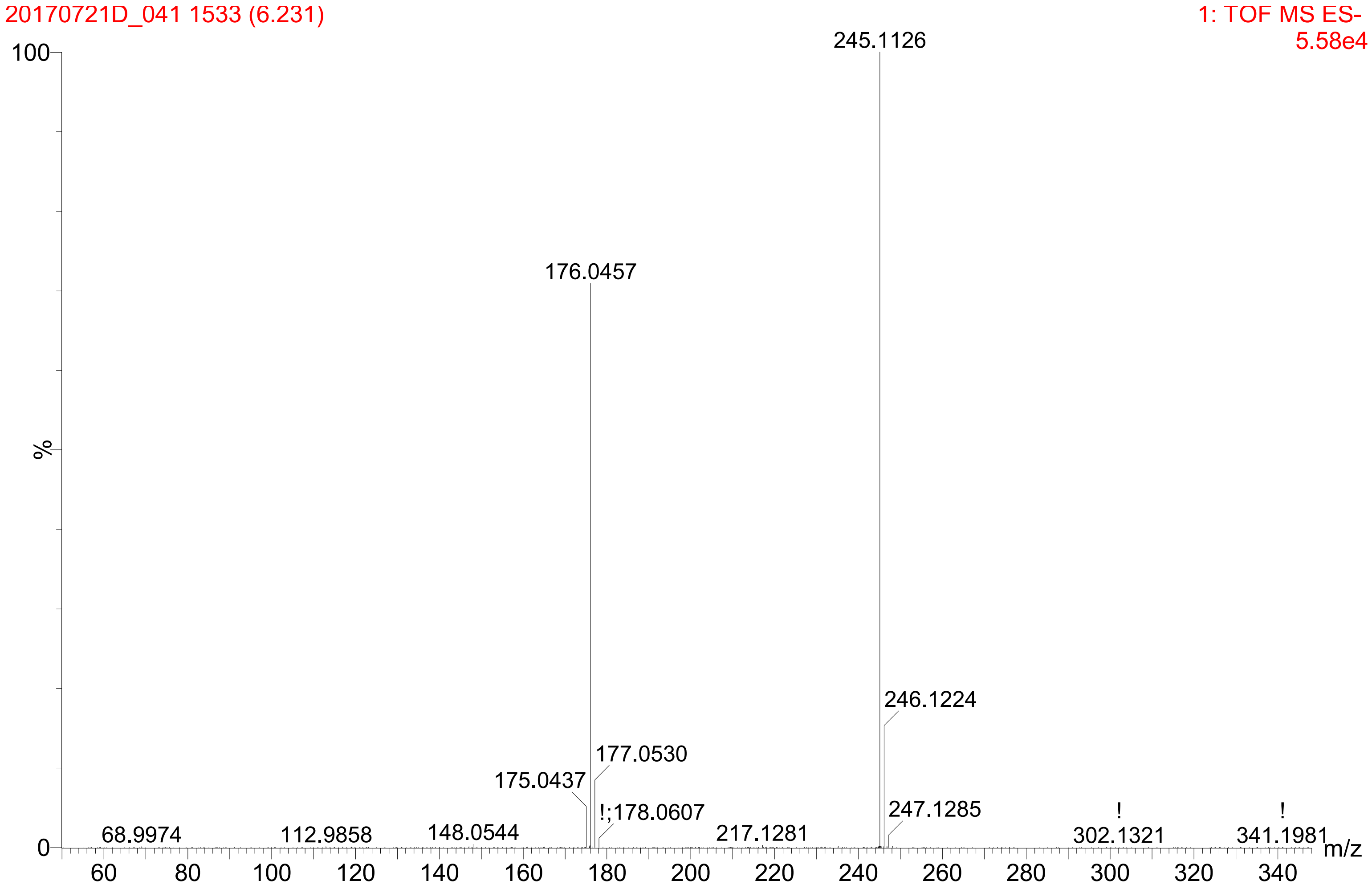

Figure S24 - HRESIMS spectrum for **Compound 4**.

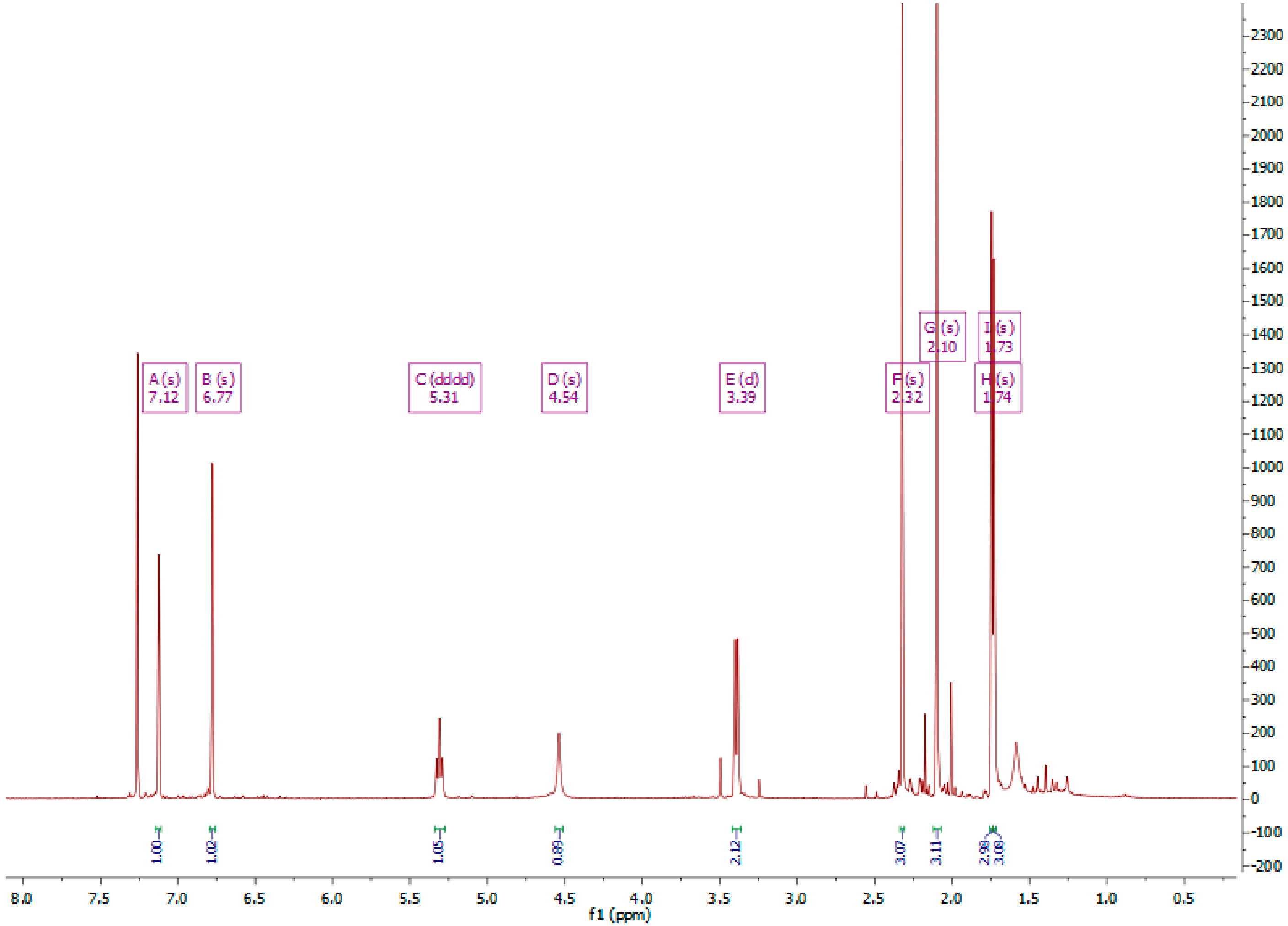

Figure 25 – ^{1}H-NMR spectrum for **Compound 5** (400 MHz, $CDCl_3$).

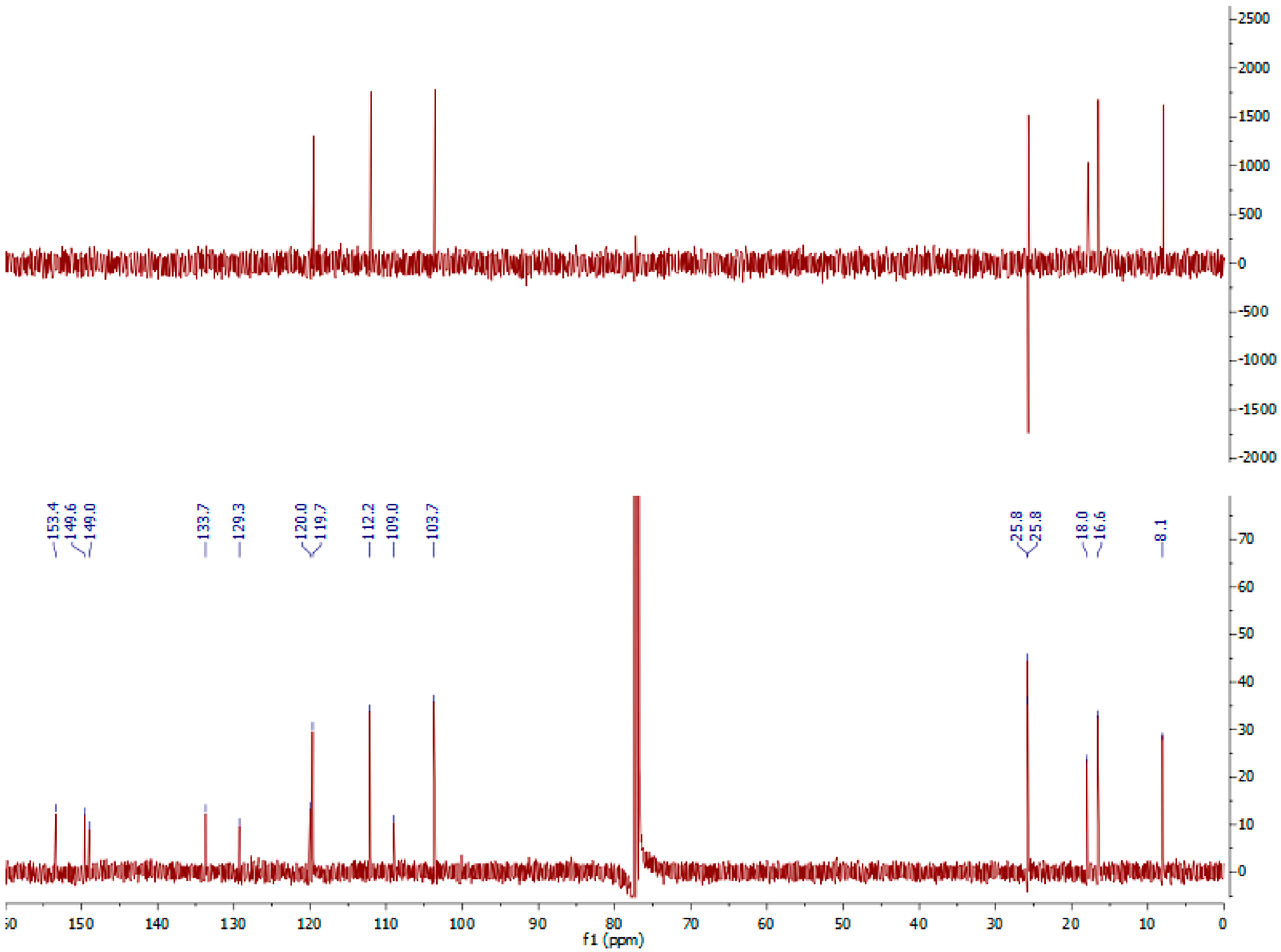

Figure 26 – DEPT and ^{13}C-NMR spectra for **Compound 5** (100 MHz, $CDCl_3$).

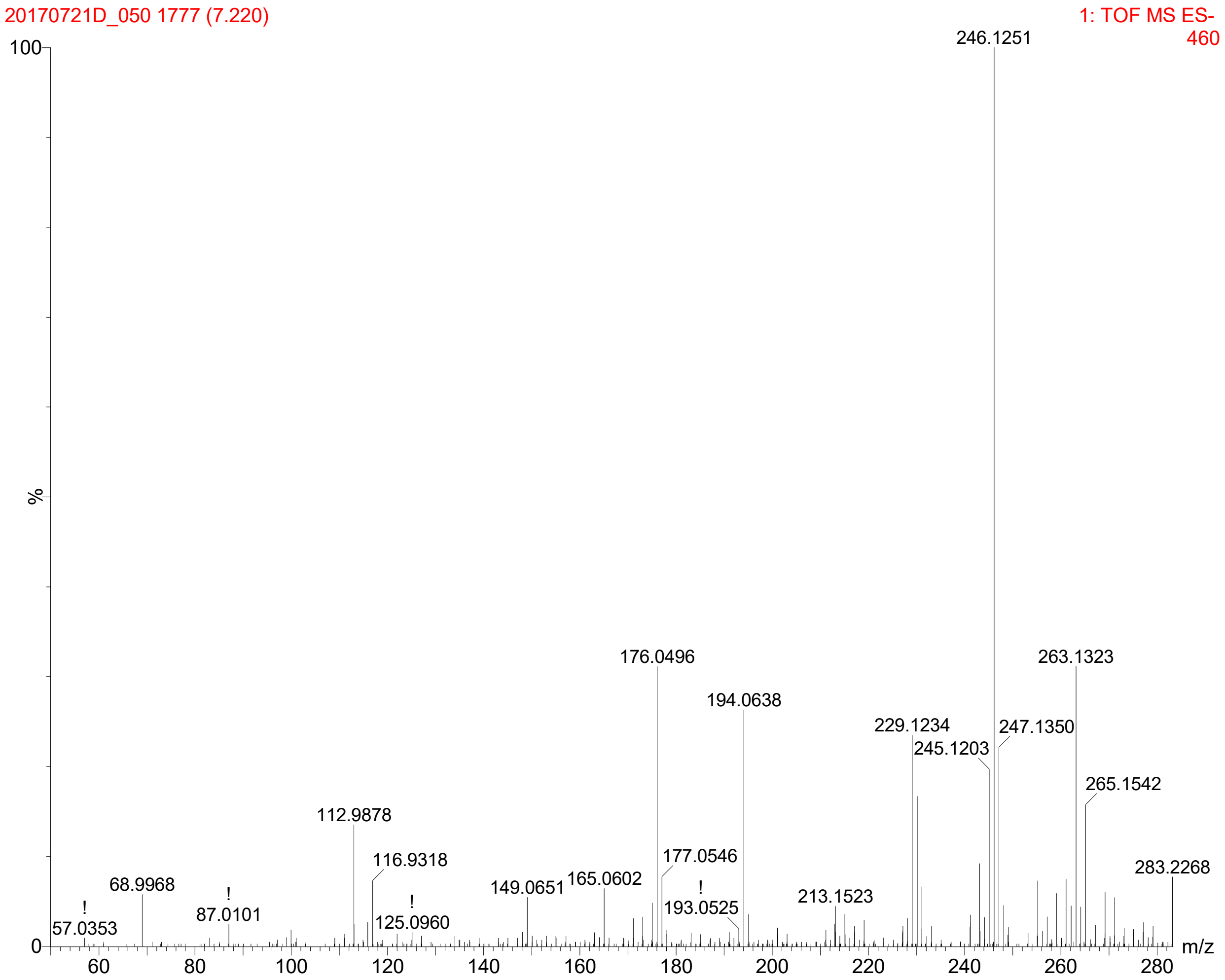

Figure S27 - HRESIMS spectrum for **Compound 5**.

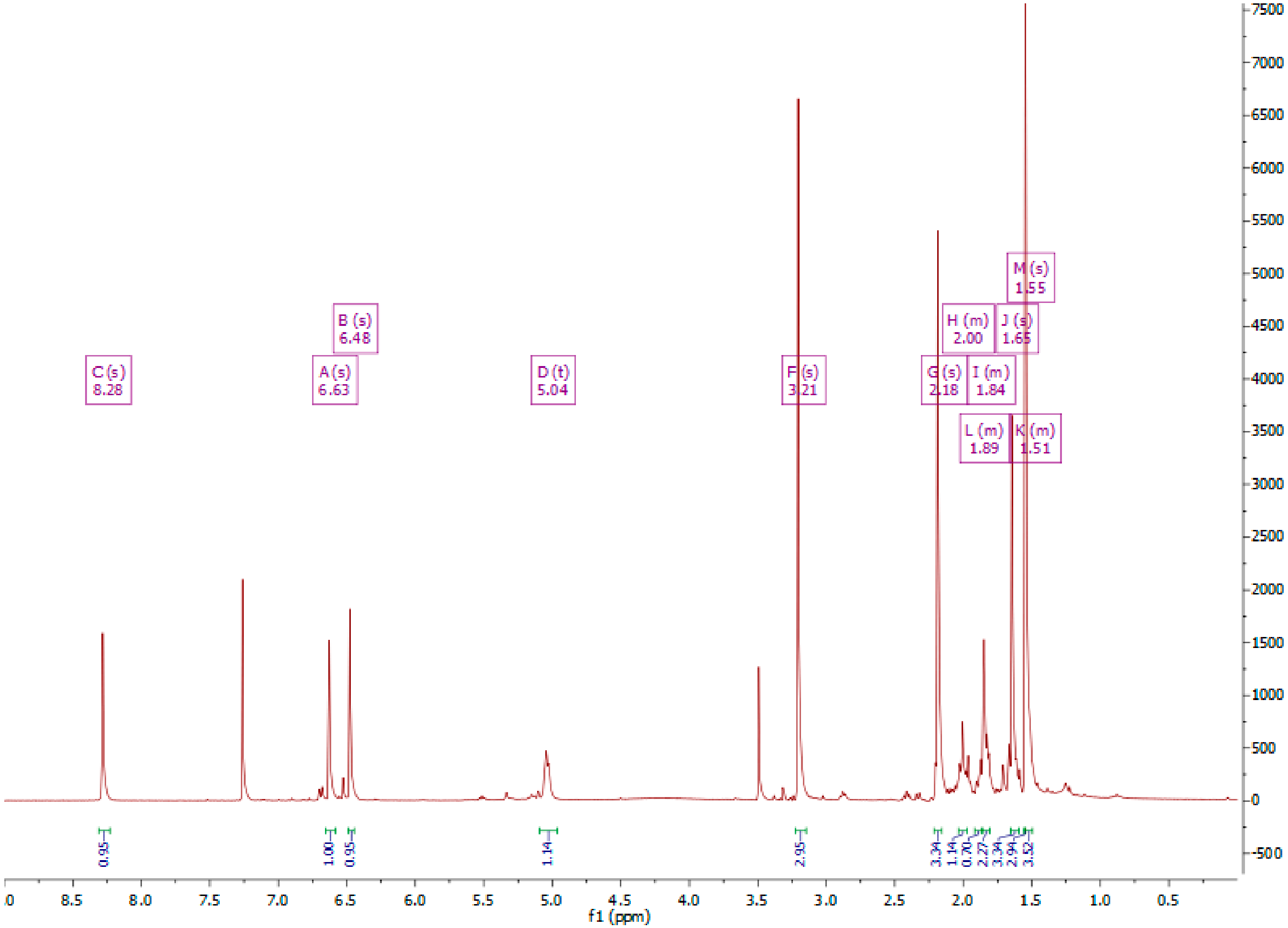

Figure S28 – ^{1}H-NMR spectrum for **Compound 6** (400 MHz, CD_3OD).

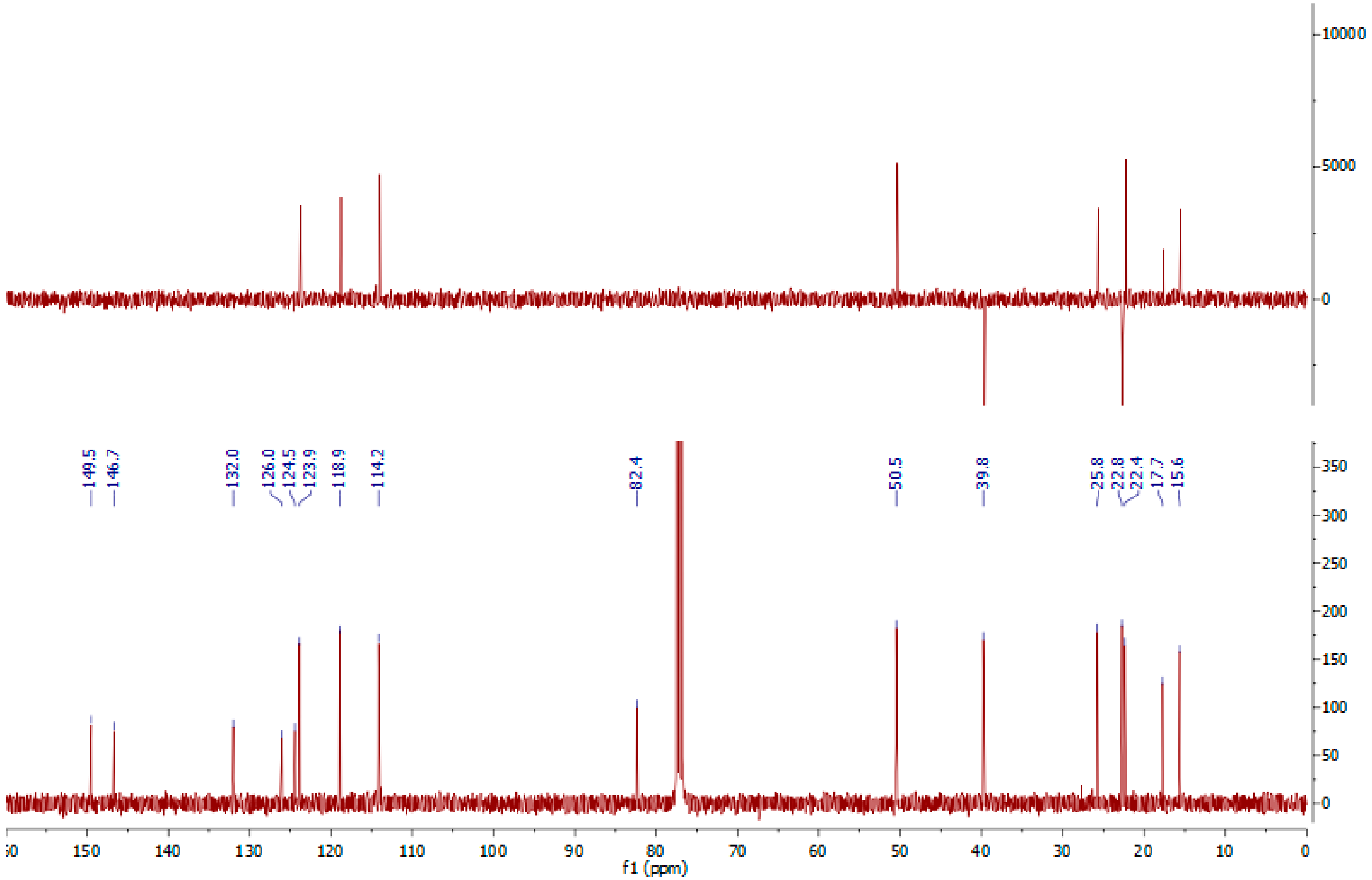

Figure S29 – DEPT and ^{13}C-NMR spectra for **Compound 6** (100 MHz, CD_3OD).

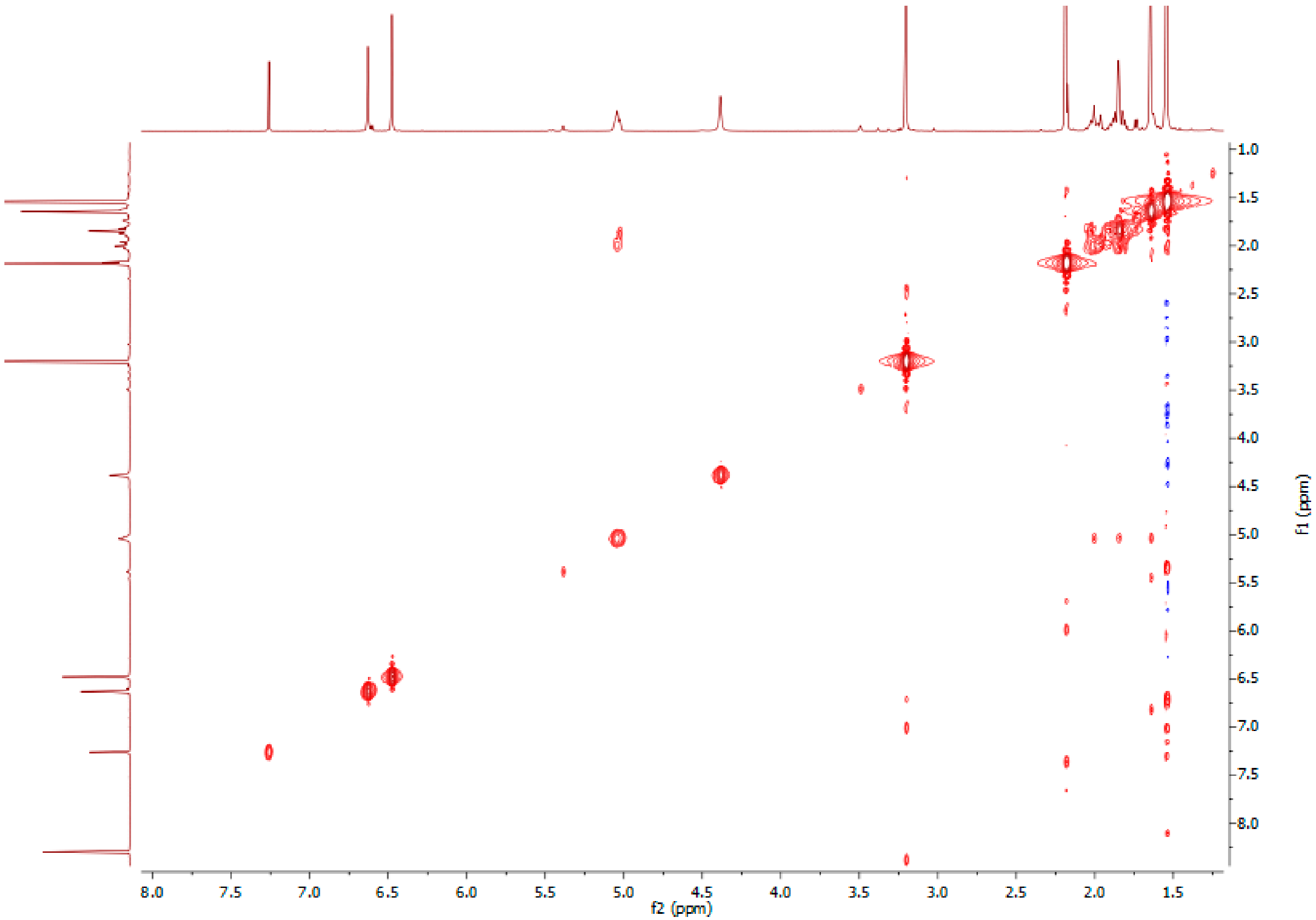

Figure S30 - *g*-COSY spectrum for **Compound 6** (400 MHz, CD_3OD).

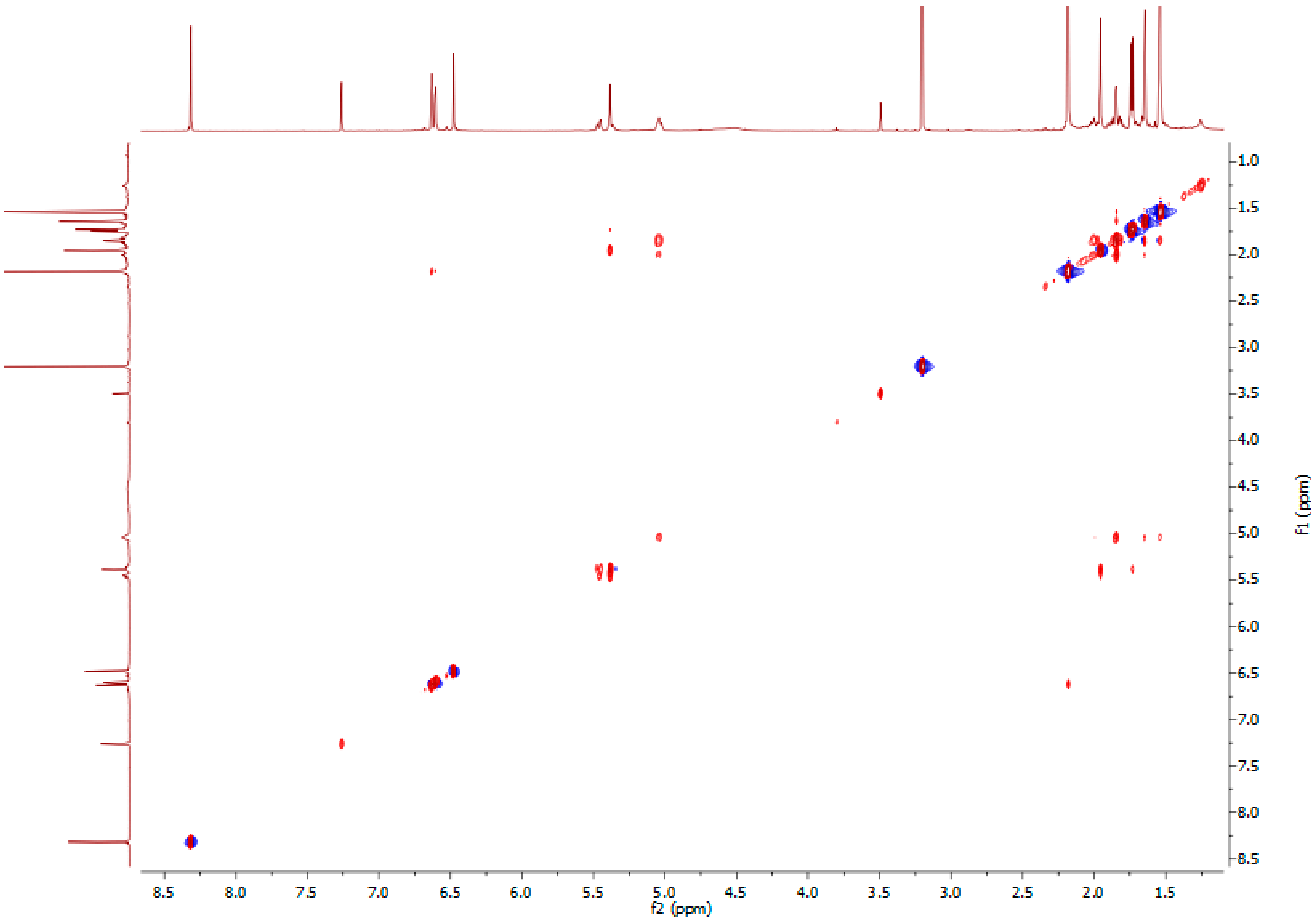

Figure S31 - TOCSY spectrum for **Compound 6** (400 MHz, CD_3OD).

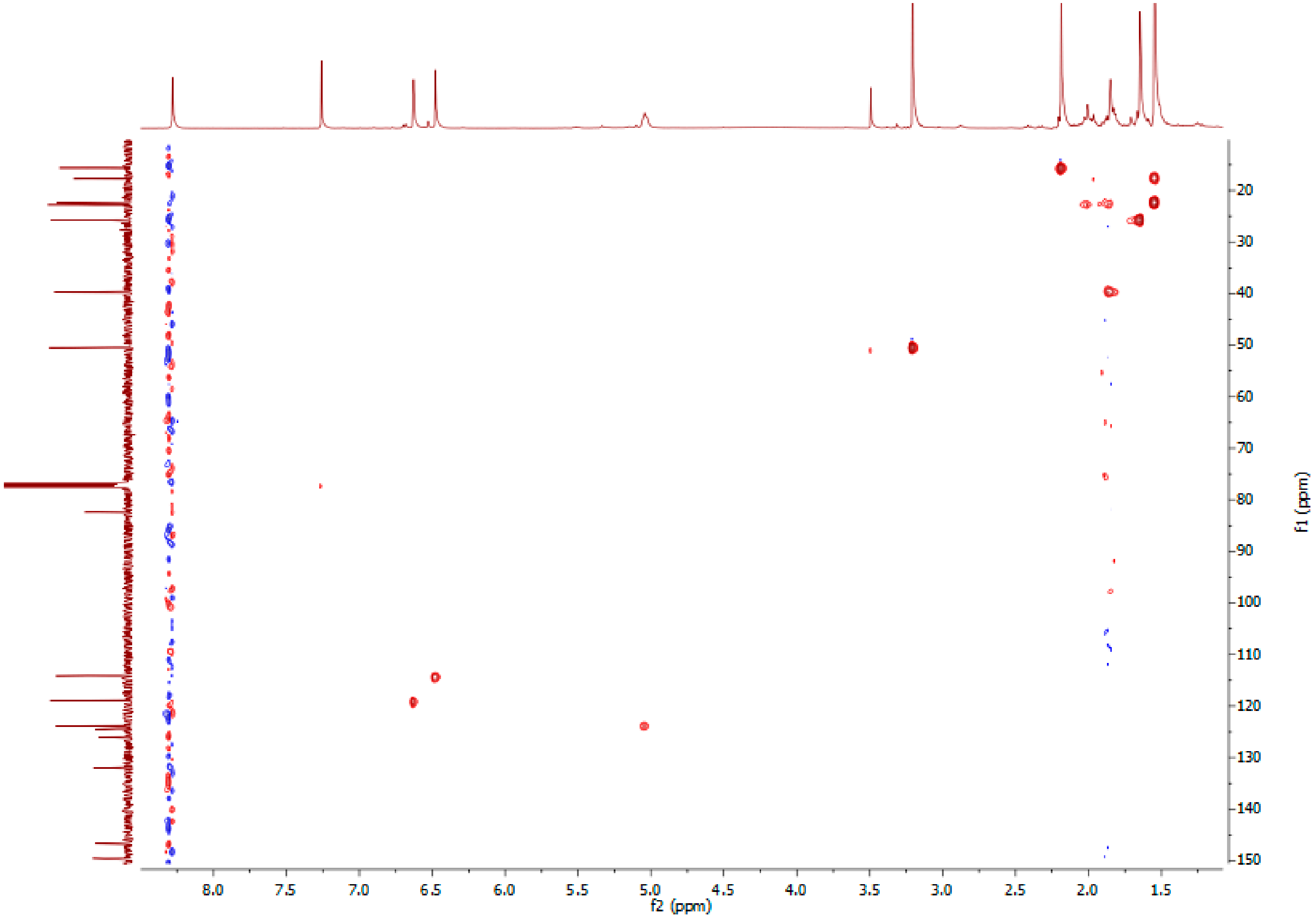

Figure S32 - *g*-HMQC spectrum for **Compound 6** (400 MHz, CD_3OD).

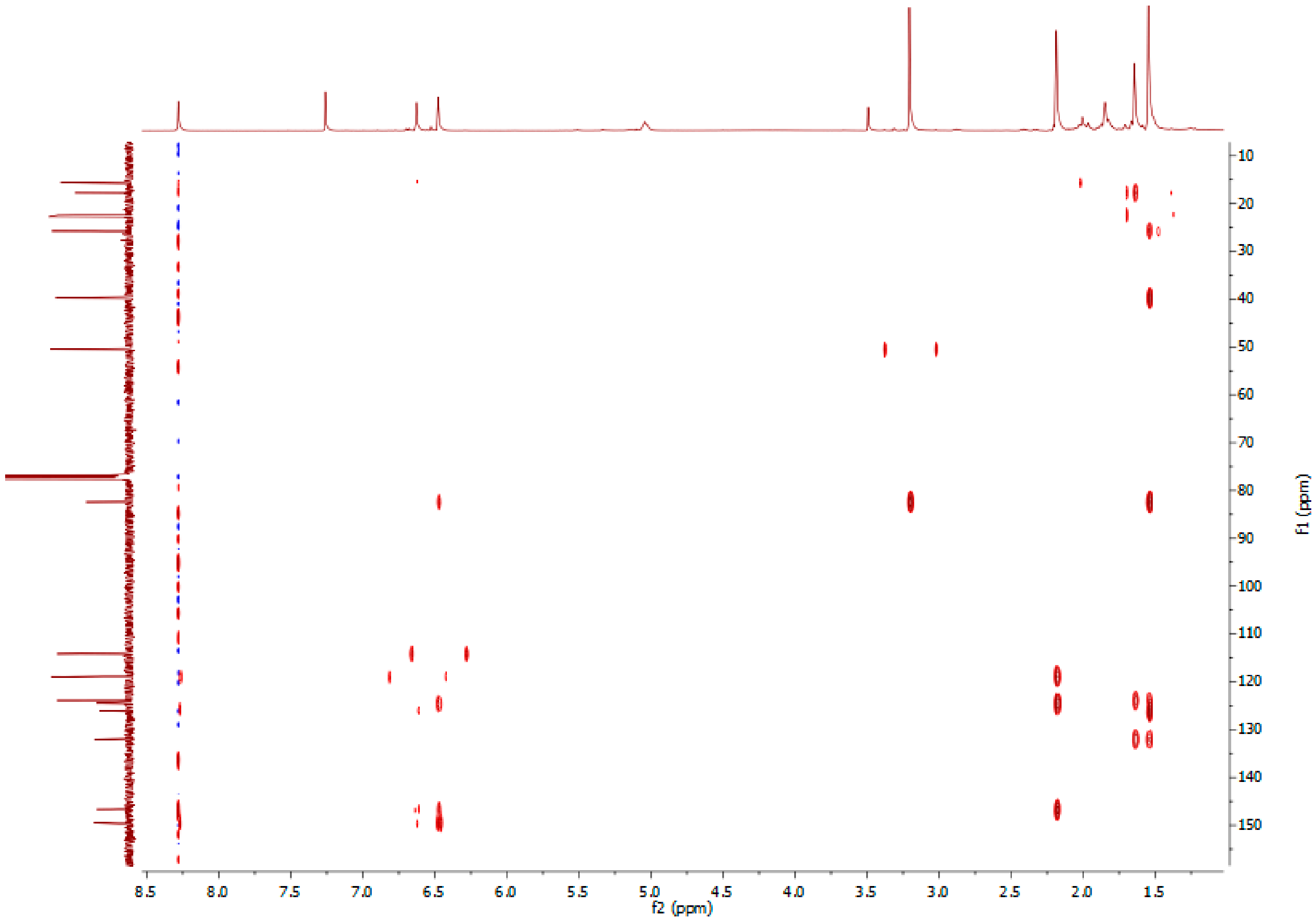

Figure 33 - *g*-HMBC spectrum for **Compound 6** (400 MHz, CD_3OD).

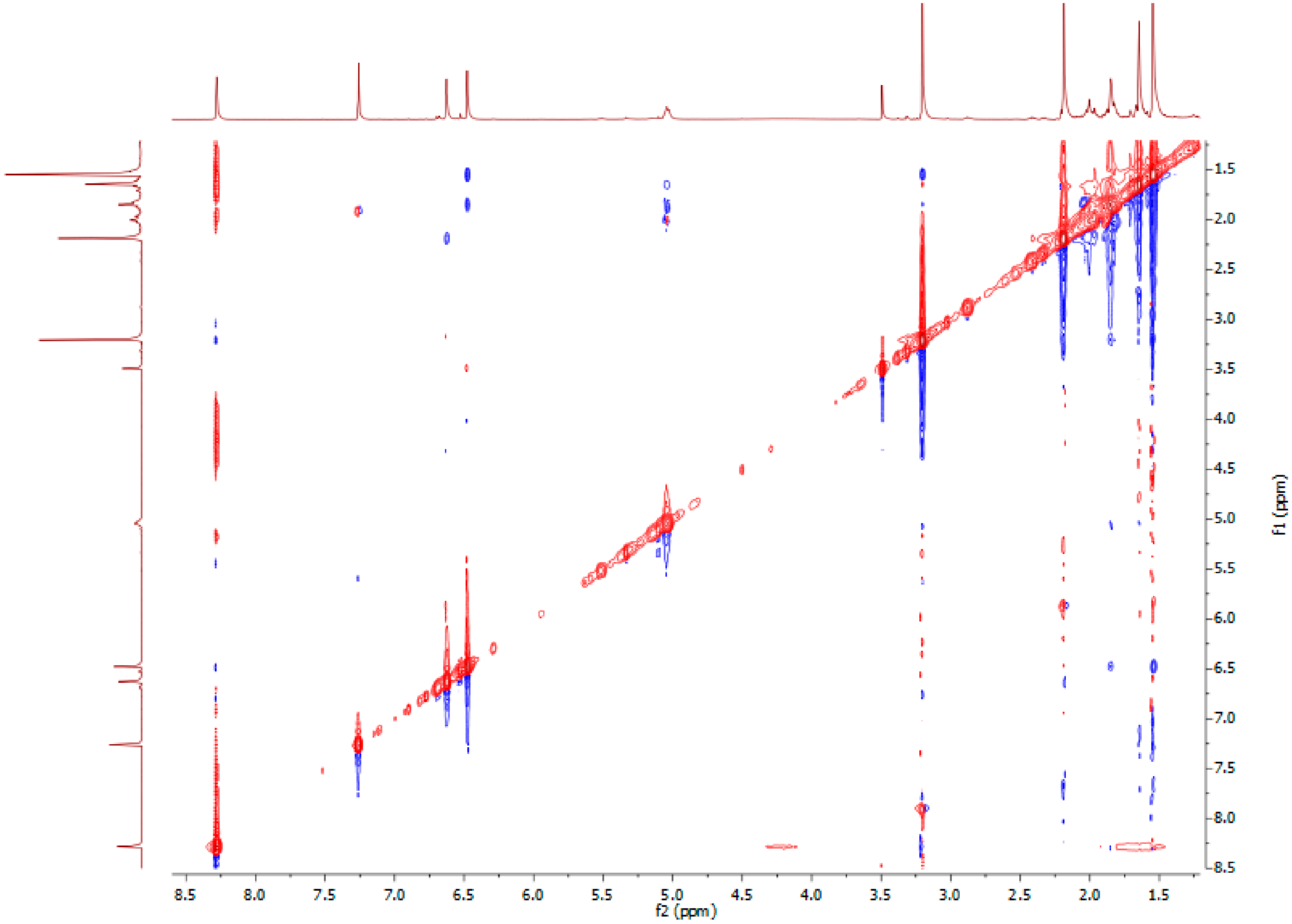

Figure 34 - NOESY spectrum for **Compound 6** (400 MHz, CD_3OD).

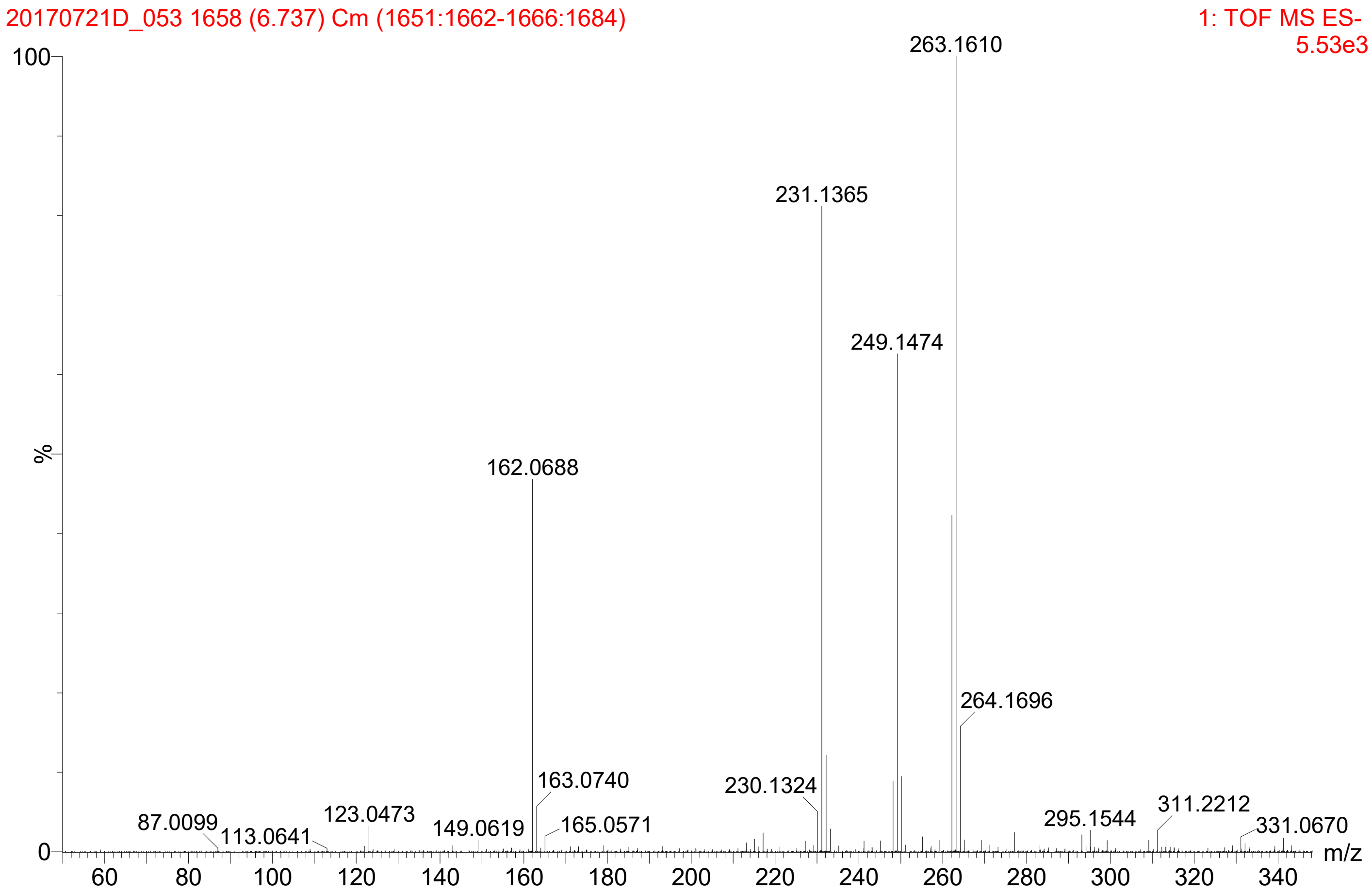

Figure S35 - HRESIMS spectrum for **Compound 6**.

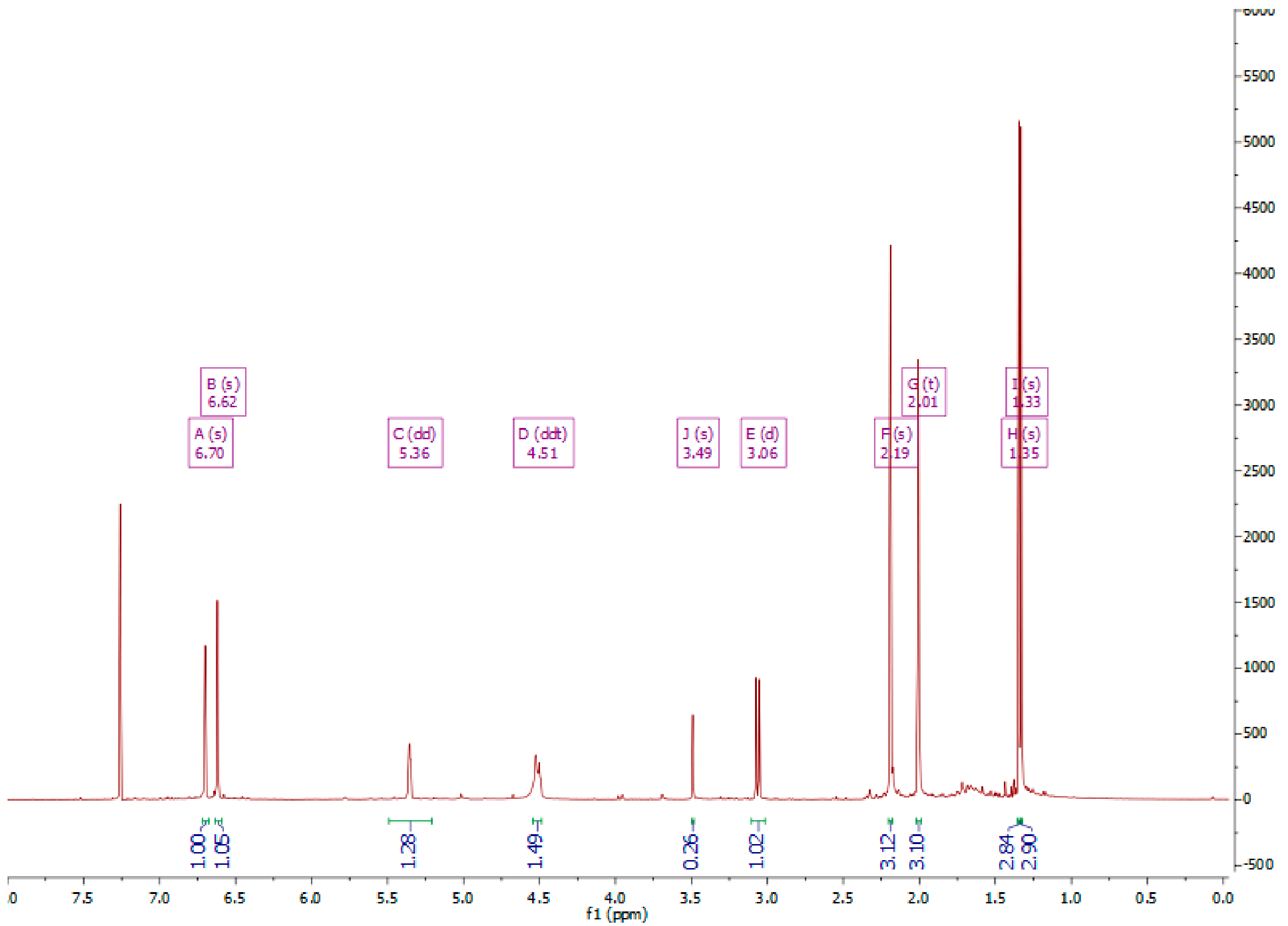

Figure S36 – ^{1}H-NMR spectrum for **Compound 7** (400 MHz, $CDCl_3$).

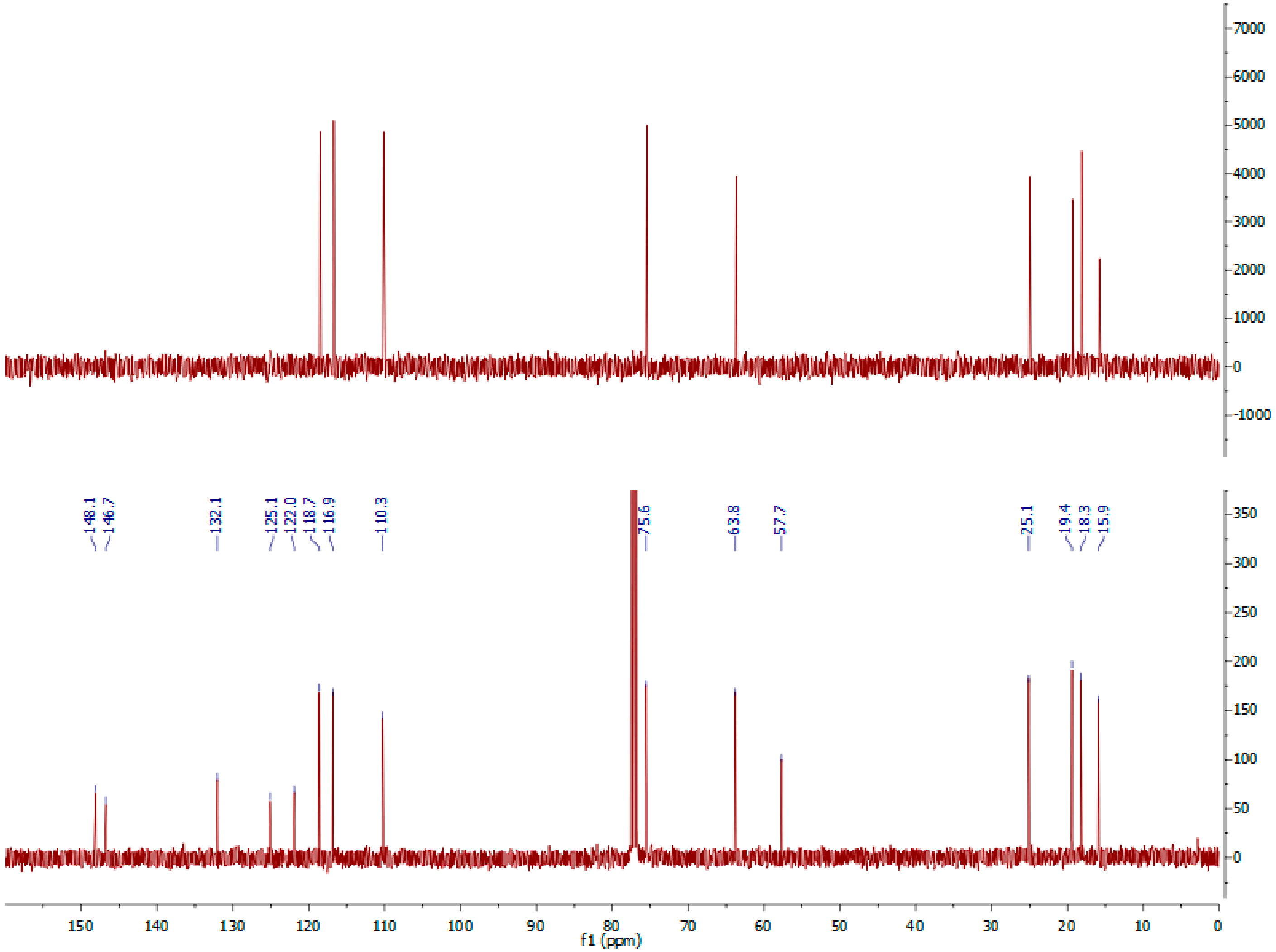

Figure S37 – ^{13}C-NMR spectrum for **Compound 7** (400 MHz, $CDCl_3$).

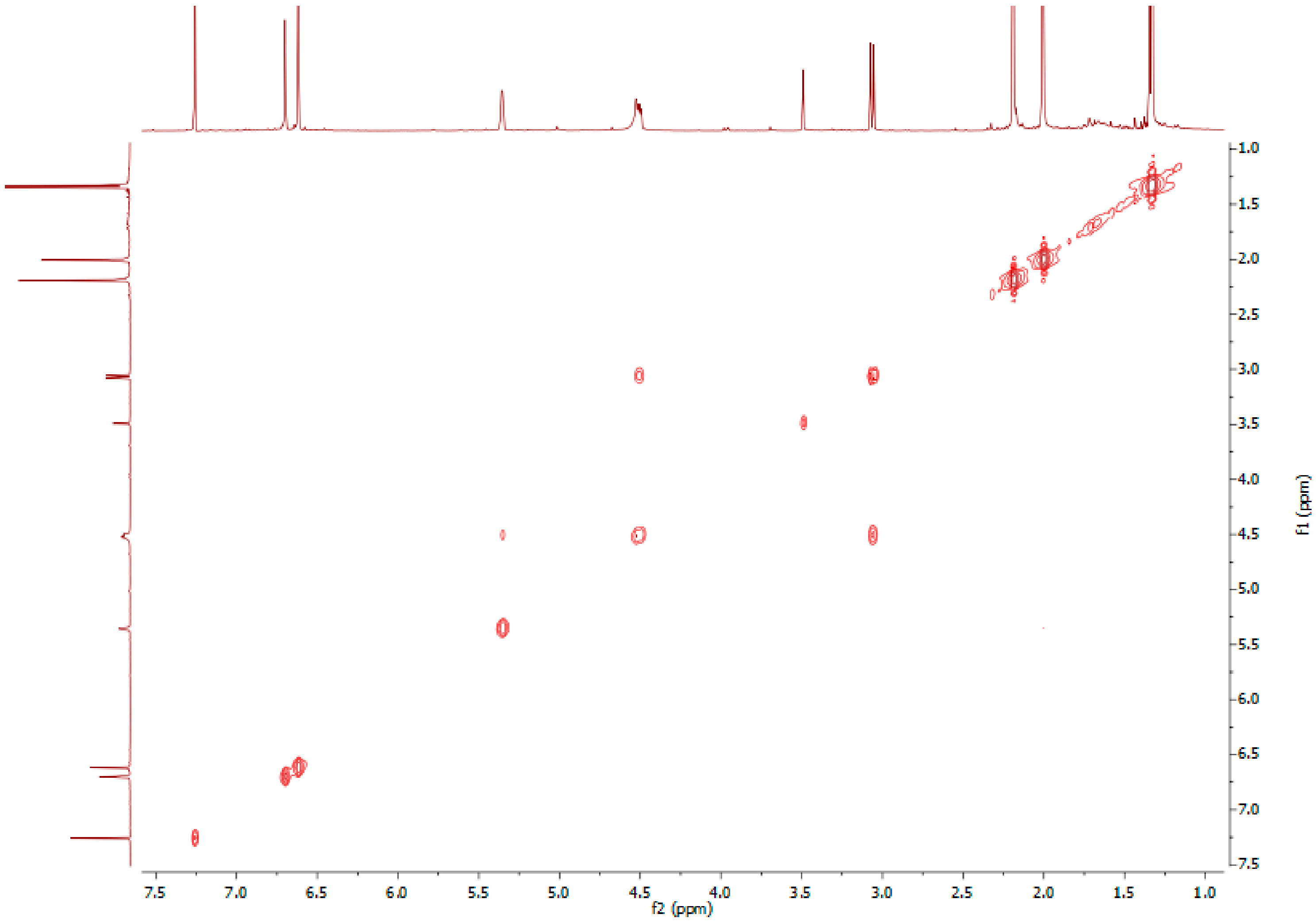

Figure S38 - *g*-COSY spectrum for **Compound 7** (400 MHz, $CDCl_3$).

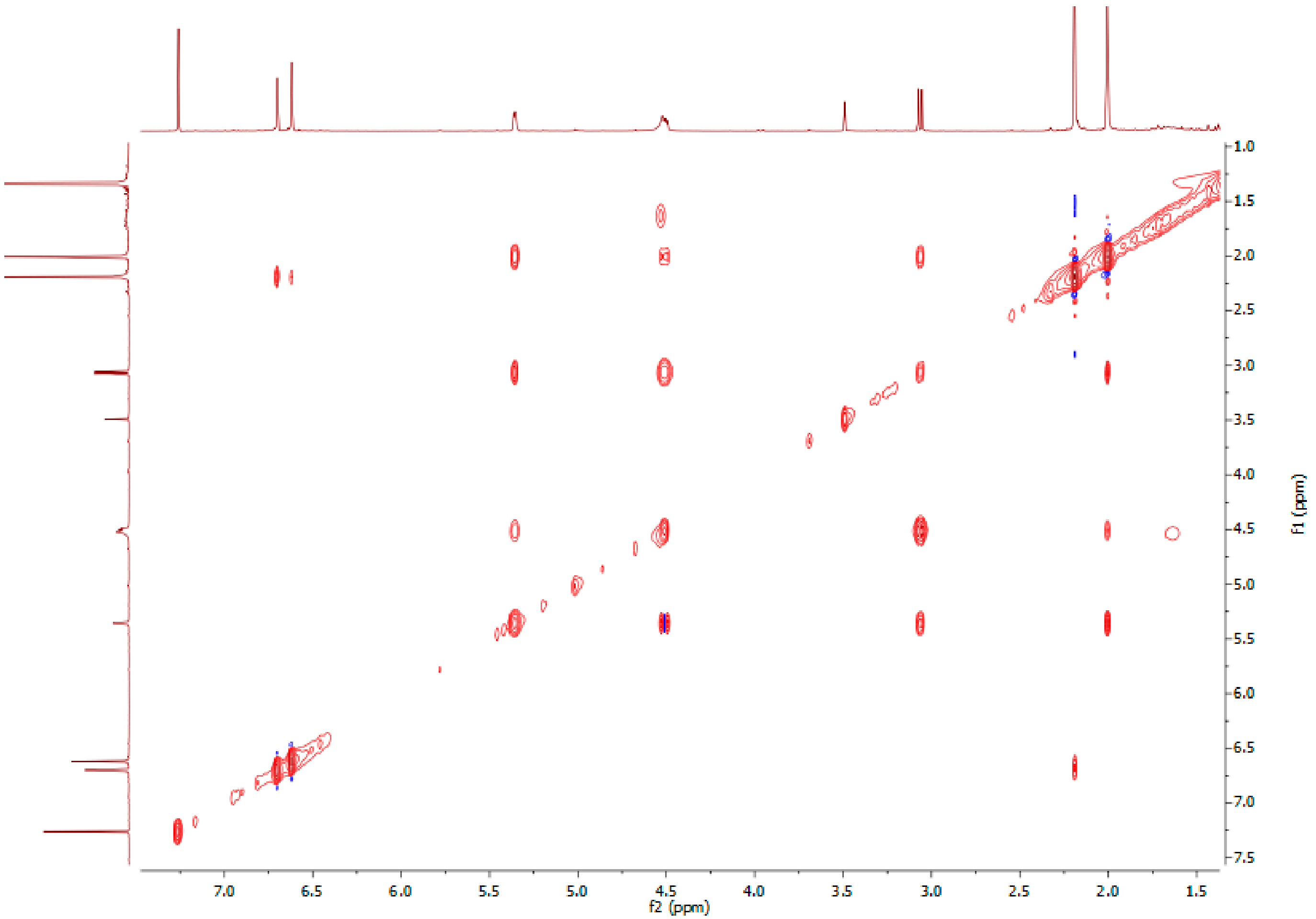

Figure S39 - TOCSY spectrum for **Compound 7** (400 MHz, $CDCl_3$).

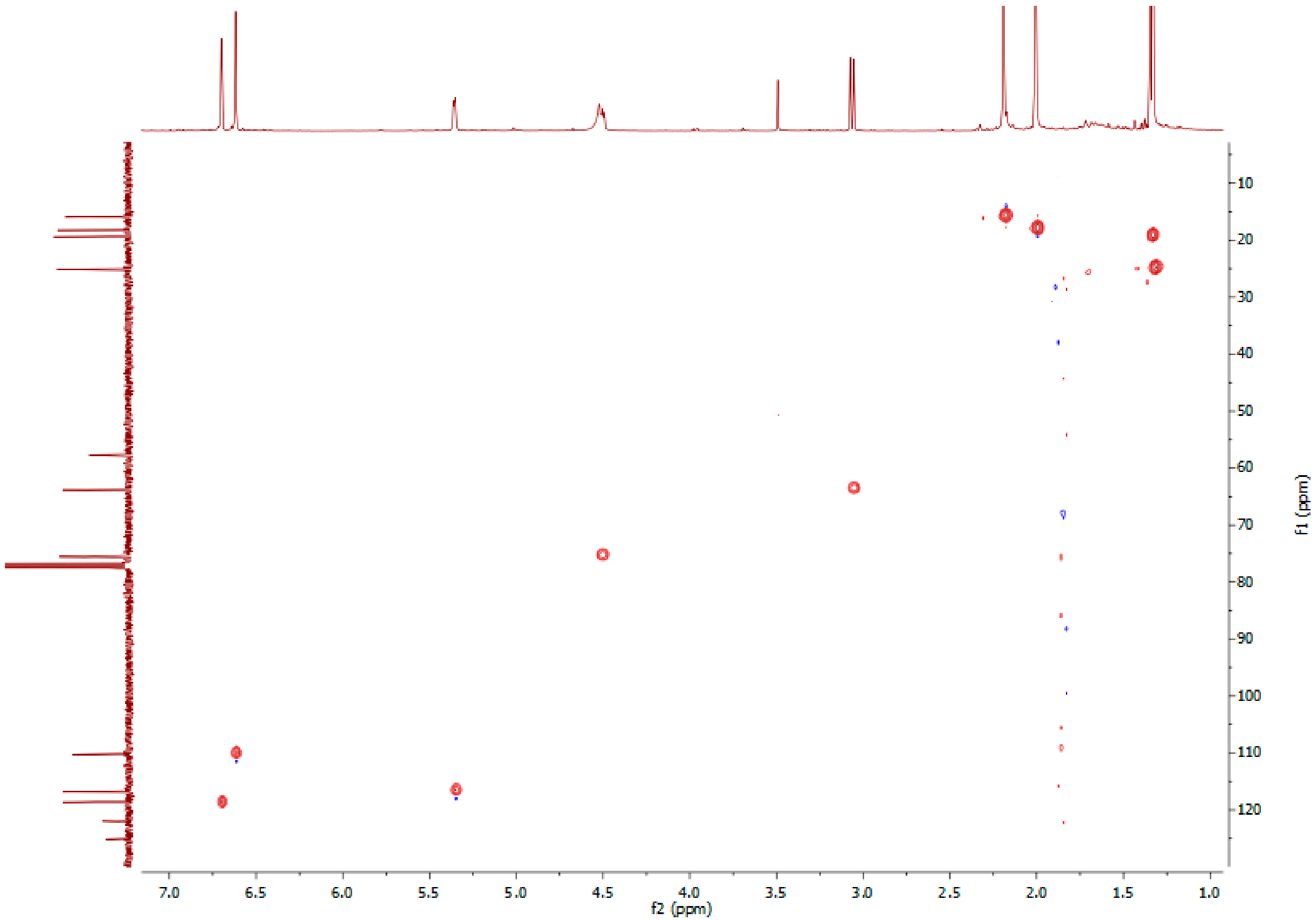

Figure S40 - *g*-HMQC spectrum for **Compound 7** (400 MHz, $CDCl_3$).

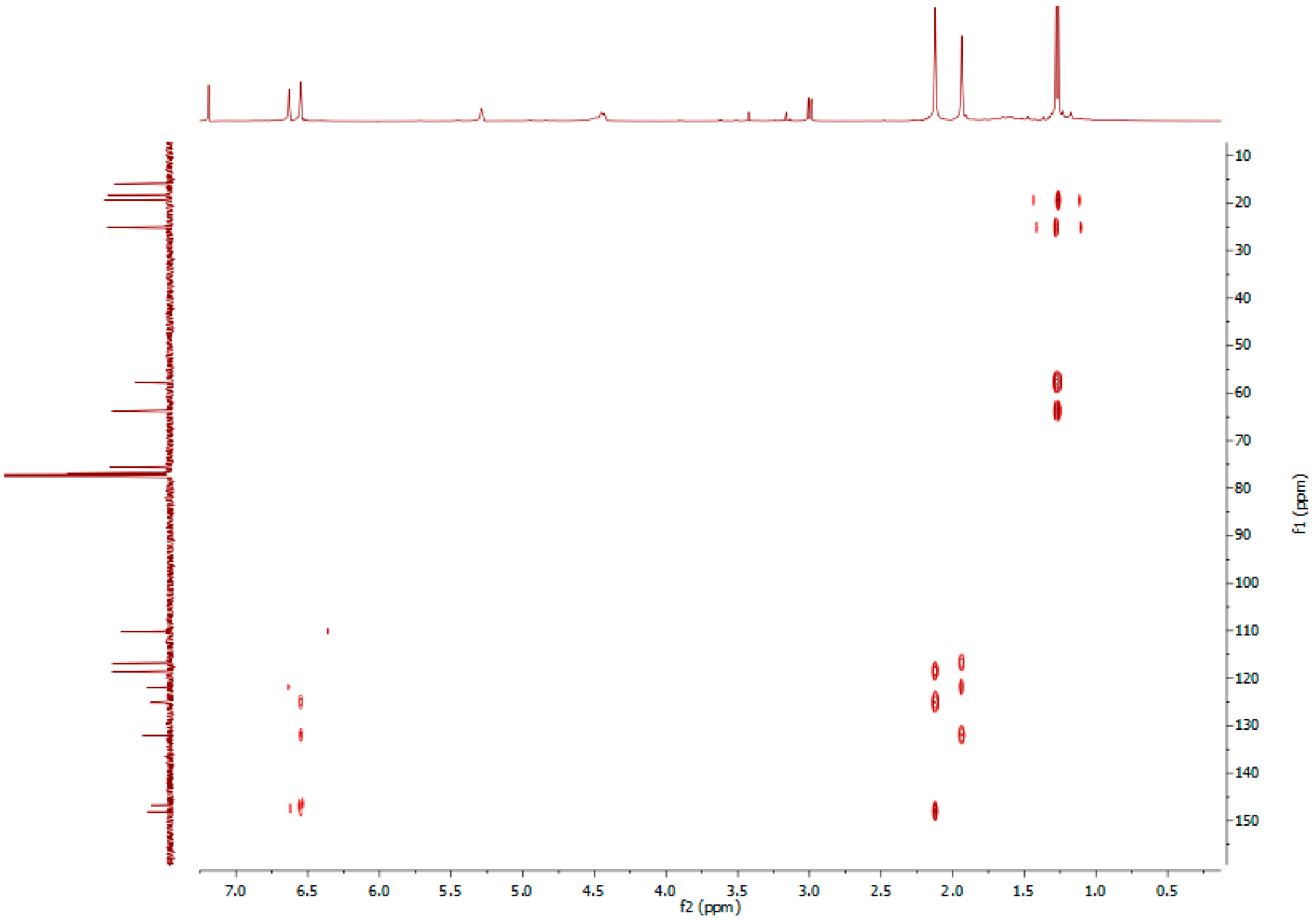

Figure S41 - *g*-HMBC spectrum for **Compound 7** (400 MHz, $CDCl_3$).

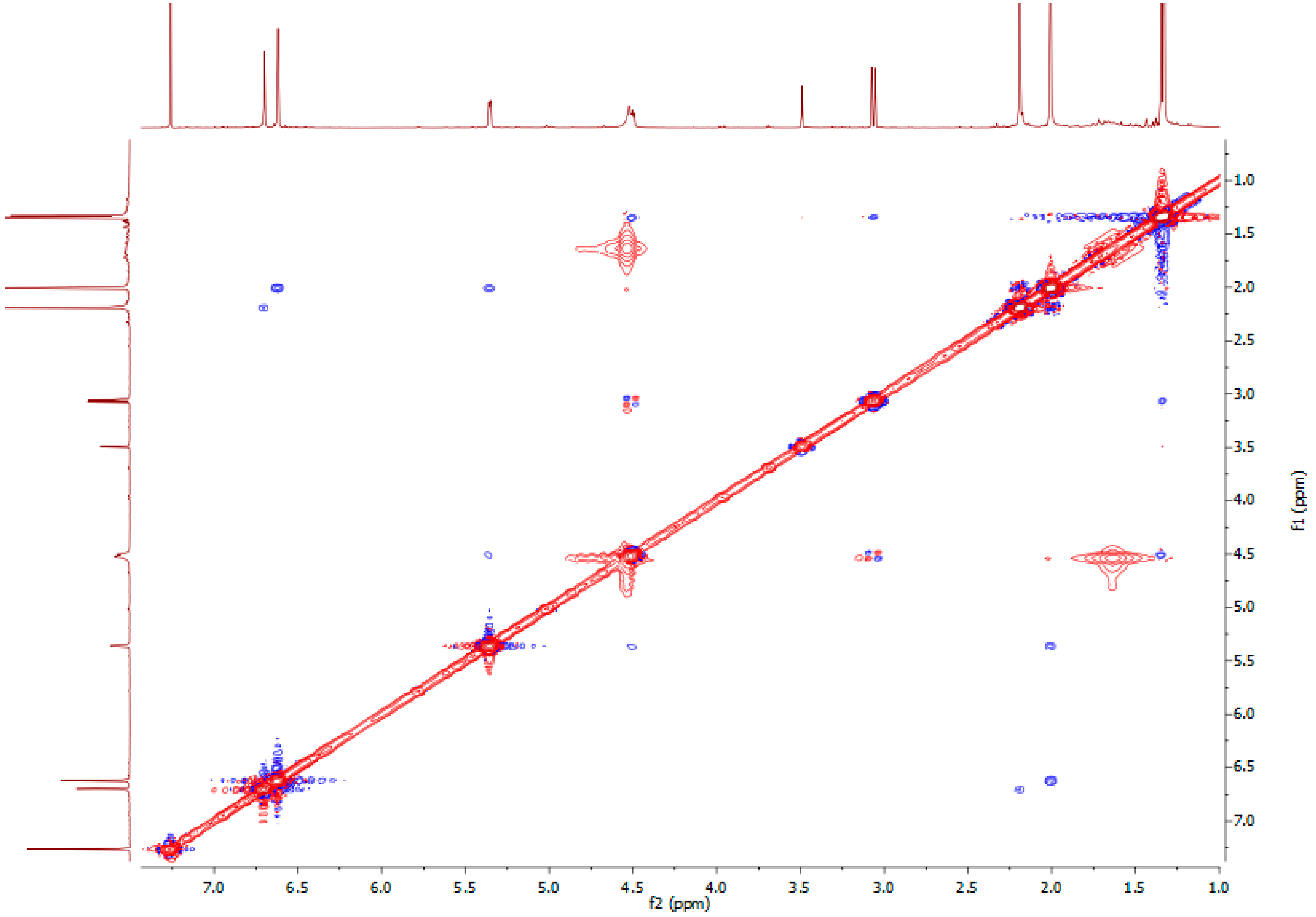

Figure S42 - NOESY spectrum for **Compound 7** (400 MHz, $CDCl_3$).

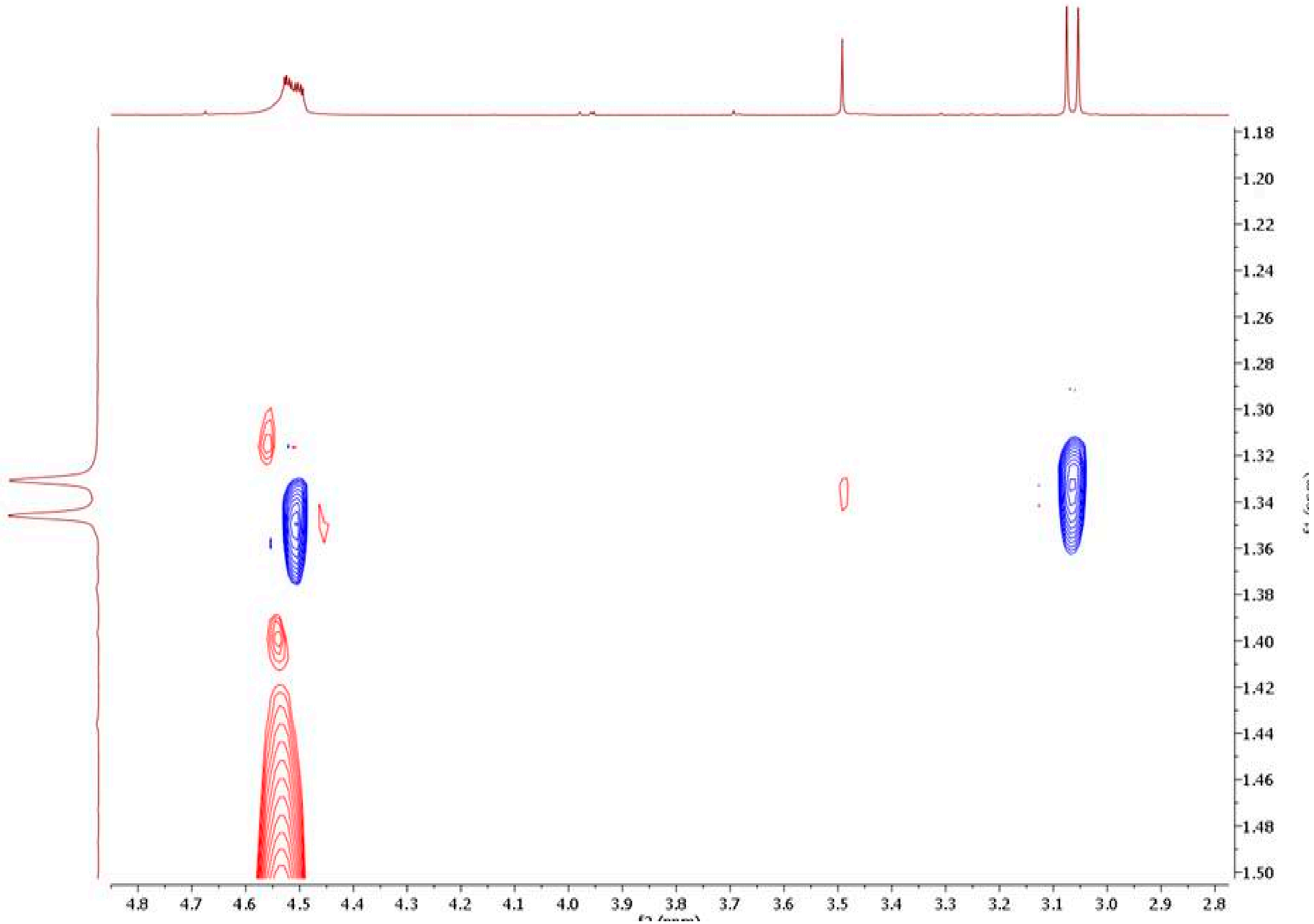

Figure S43 – Close up of NOESY spectrum for **Compound 7** (400 MHz, $CDCl_3$).

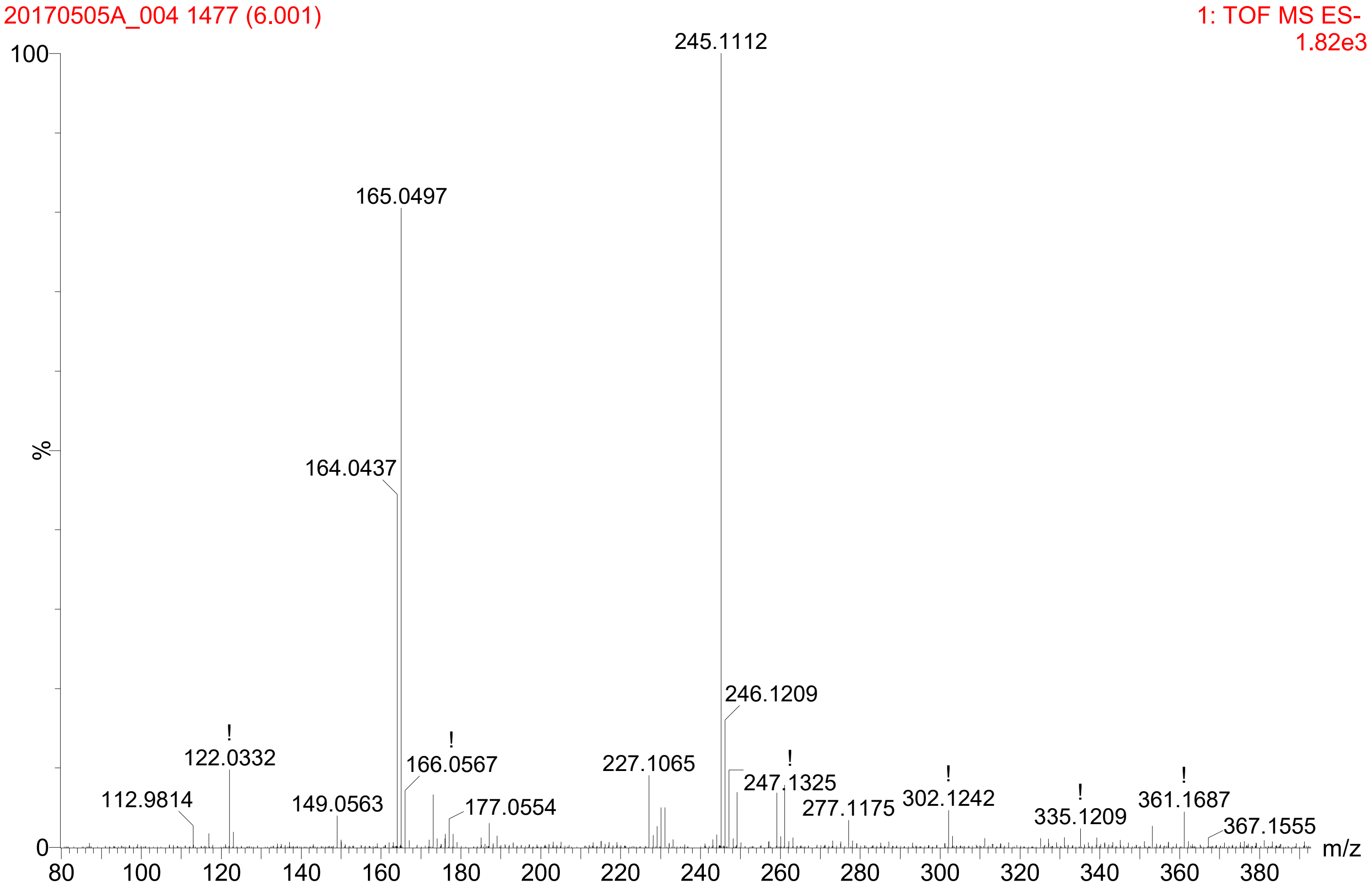

Figure S44 - HRESIMS spectrum for **Compound 7**.

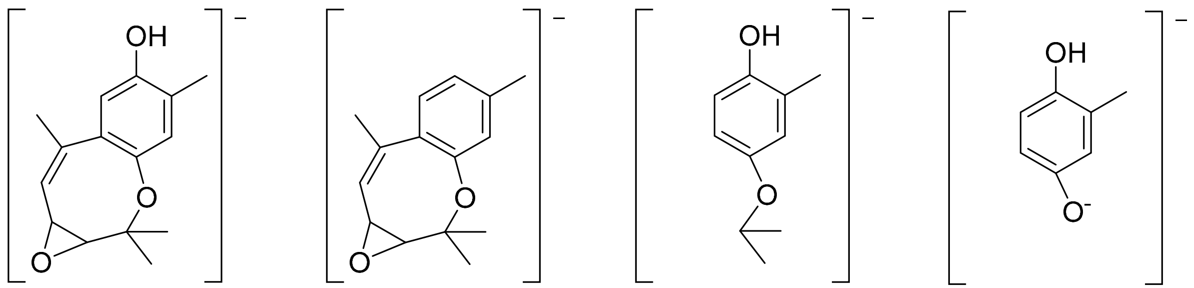

m/z 245.1234
calculated 245.1177

m/z 230.1421
calculated 230.1367

m/z 165.0497
calculated 165.0915

m/z 122.0332
calculated 165.0368

Figure S45 - Compound 7 MS fragmentation ions induced by ESI (negative ionization mode).
